# Microglial NLRP3-dependent pyroptosis promotes cognitive dysfunction of diabetic encephalopathy by inhibiting adult hippocampal neurogenesis through the release of IL-1β

**DOI:** 10.1038/s41401-026-01774-0

**Published:** 2026-03-20

**Authors:** Meng-yu Hua, Shan Huang, Zi-yun Zhuang, Xiao-lin Han, Xiao-jing Liu, Zhong-hao Liang, Neng-jun Lou, Feng-jie Zheng, Li lv, Xiang-hua Zhuang, Shu-yan Yu, Shi-hong Chen

**Affiliations:** 1 Department of Endocrinology and Metabolism, The Second Qilu Hospital of Shandong University, Jinan, 250033 China; 2 Department of Endocrinology and Metabolism, The First People’s Hospital of Jinan, Jinan, 250011 China; 3 Multidisciplinary Innovation Center for Nephrology of the Second Qilu Hospital of Shandong University, Jinan, 250033 China; 4 Department of Physiology, School of Basic Medical Sciences, Cheeloo College of Medicine, Shandong University, Jinan, 250012 China

**Keywords:** NLRP3 inflammasome, IL-1β, pyroptosis, adult hippocampal neurogenesis, diabetic encephalopathy, cognitive dysfunction

## Abstract

Diabetic encephalopathy (DE) is a prevalent complication of diabetes which can lead to cognitive dysfunction, without effective therapy currently. In diabetic patients, a reduction in adult hippocampal neurogenesis (AHN) is a heightened risk of cognitive impairment, which may be associated with neuroinflammation caused by microglia. In this study, we established a DE mouse model and conducted in vitro cultures of microglial cells and neural stem cells. Our study demonstrated that the high-glucose associated with DE impairs AHN and induces microglial NOD-, LRR-, and pyrin domain-containing protein 3 (NLRP3) dependent pyroptosis. Further investigation showed that upregulation of microglial NLRP3 promotes the activation of Gasdermin D (GSDMD), the key pyroptosis effector, and the cleavage of pro-interleukin-1β (pro-IL-1β) by caspase-1, exacerbated pyroptosis and induced release of IL-1β, which might lead to impaired AHN and subsequent cognitive dysfunction. Conversely, downregulation of microglial NLRP3 inhibited caspase-1 activation and pyroptosis, reduced release of IL-1β, improved AHN, and rescued cognitive deficits in DE mouse model. Such findings suggest that targeting microglial NLRP3 inflammasome-mediated pyroptosis may be an important potential therapeutic target for treating DE.

**Figure:**
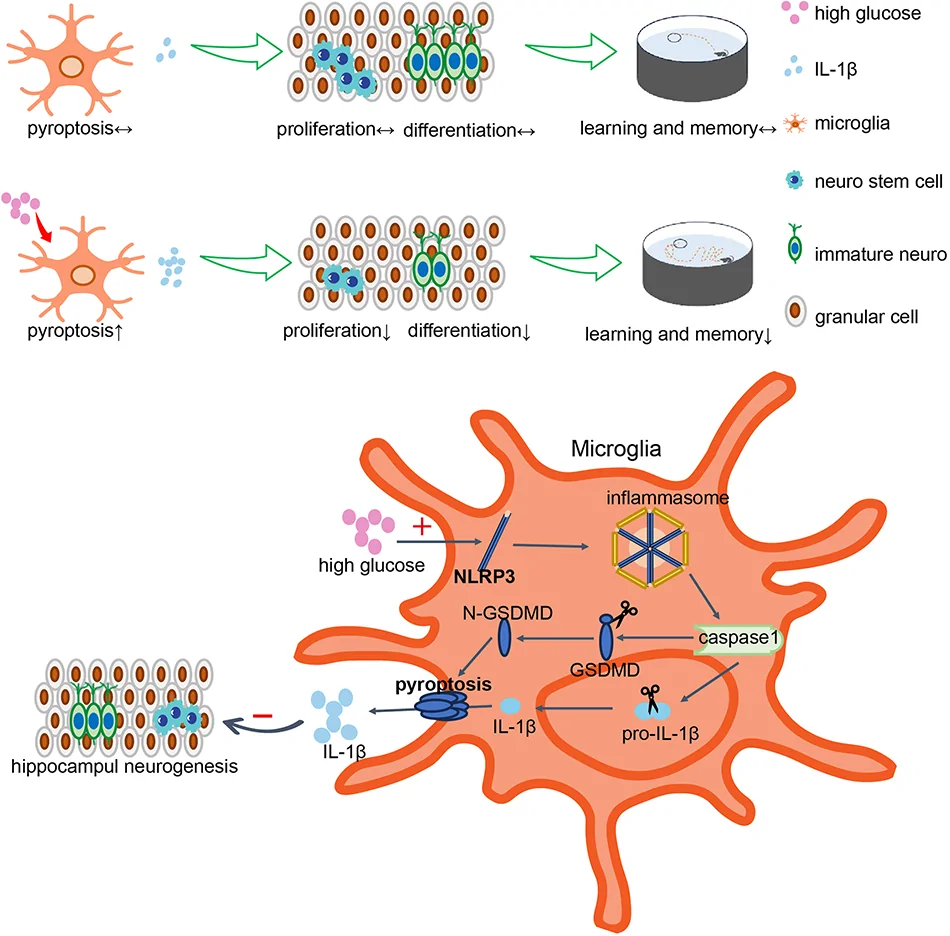

## Introduction

Diabetic encephalopathy (DE) is a significant complication arising from diabetes which can alter central nervous system functions. DE manifests as behavioral and cognitive changes, accompanied by neurochemical and structural alterations within the brain, with the hippocampus being notably affected [1, 2]. Although the concept of DE was initially proposed by Nielsen in the 1960s [3], the pathophysiological mechanisms remain to be fully elucidated. There is evidence indicating that individuals with diabetes are at a higher risk for cognitive impairment, a behavioral deficit which correlates with reduced adult hippocampal neurogenesis (AHN) [4, 5].

AHN refers to the process in which neural stem cells (NSCs) proliferate and migrate to genesis center, then differentiate into mature neurons and granule cells [6]. It is now widely accepted as a phenomenon occurring in the subventricular zone and the dentate gyrus (DG) subgranular zone (SGZ) of the hippocampus [7]. These processes—including proliferation, differentiation, migration, neurite extension, and synaptic integration of NSCs—are governed by numerous regulatory signals, such as neuroinflammation, oxidative stress, and mitochondrial dynamics [8–10]. There is a growing consensus that AHN is essential for hippocampal function, with disruptions of AHN resulting in cognitive impairment, memory deficits, depression, and anxiety [11, 12]. Results from behavioral studies have established that a positive correlation exists between the extent of neurogenesis in rodents and their performance on hippocampal-dependent functions [13], such as mood regulation [5] and memory [4]. Interestingly, the hallmarks of diabetes—hyperglycemia and insulin resistance—have been demonstrated to impede AHN, thereby contributing to cognitive decline [14, 15]. Previous studies have demonstrated that diabetic mouse models exhibit significant cognitive deficits, accompanied by reduced AHN [16]. These findings suggested that the reduction in AHN may play a key role in cognitive impairment associated with DE. However, the molecular signaling pathway involved in the regulation of AHN in DE remains unclear. AHN is orchestrated by microglia, astrocytes and vasculature of the DG neurogenic niche [17]. In recent years, microglia have been shown to be important effectors of AHN [18, 19]. Microglia, the primary immune cells in the central nervous system, exhibit macrophage-like functions and regulate neuroinflammation and immunity [20]. Numerous literatures have demonstrated that in injury or disease states, microglia have a negative effect on AHN by releasing pro-inflammatory factors and promoting neuroinflammatory environment [21, 22].

Pyroptosis is a proinflammatory programmed cell death that is a critical step leading to the release of inflammatory factors [22, 23]. The NOD-, LRR-, and pyrin domain-containing protein 3 (NLRP3) inflammasome is a key molecular complex that orchestrates innate immune responses [24]. Micro infection, endogenous danger signals and environmental stimuli promote the activation of the NLRP3 inflammasome, which induces the self-cleavage of pro-caspase-1 into the active caspase 1-p20, leading to the maturation of the pro-inflammatory cytokine interleukin 1β (IL-1β) and interleukin 18 (IL-18). Caspase-1-p20 also cleaves gasdermin D (GSDMD) and releases its N-terminal domain, N-GSDMD is transferred to the cell membrane and forms pores, mediating the release of inflammatory factors IL-1β and IL-18, and inducing inflammatory cell death, known as pyroptosis [25, 26]. Upon brain injury or harmful stimuli, NLRP3 inflammasomes in microglia are activated, leading to pyroptosis and the release of proinflammatory cytokines associated with neurodegenerative diseases [27]. In a mouse model of neuropsychiatric lupus, it was found that the activation of microglia leads to the release of pro-inflammatory factors, resulting in the disruption of hippocampal neurogenesis [19]. Integrating the aforementioned evidence, we have identified that under the condition of central nervous system diseases, microglial pyroptosis exacerbates neuroinflammation and inhibits AHN through the release of pro-inflammatory factors. Whether NLRP3-dependent pyroptosis of microglial and the release of IL-1β lead to neurogenesis disorders in DE requires experimental verification.

Given the rising prevalence of diabetes and the substantial impact of DE on patients’ quality of life, it is imperative to elucidate the pathogenesis of DE and identify potential therapeutic strategies. By establishing DE and targeted expression regulation of microglial NLRP3 models, we found that microglial NLRP3 plays a critical role in the regulation of AHN and the associated cognitive impairments in DE mice by modulating pyroptosis and the release of IL-1β.

## Materials and methods

### Animals care

Male C57BL/6J mice, 6 weeks of age, obtained from the Beijing Vital River Laboratory Animal Technology Co., Ltd (Beijing, China) were used in these experiments. All mice were housed individually in ventilated cages, at a temperature maintained at 24 ± 2 °C and under a 12 h–12 h light–dark cycle in the animal laboratory of the Second Hospital of Shandong University. The mice were provided with unrestricted access to food and water for the entire duration of the experiment. During the entire experimental period, the health of these mice was continuously monitored to reduce the number of mice used in the experiments as well as to alleviate their stress and suffering. All animal experiments were performed in accordance with the National Institutes of Health Guide for the Care and Use of Laboratory Animals (National Research Council, 1996) and were approved by the Research Ethics Committee of the Second Hospital of Shandong University (Approval No. KYLL-2023LW120).

### In vivo diabetes model

Prior to use in the experiments, mice were allowed to adapt to laboratory conditions for 1 week. They were then randomly divided into two groups. One group was exposed to a 6-week high-fat diet (HFD) followed by injections of streptozotocin (STZ, #S8050, Solarbio, Beijing, China) to generate T2DM model. The HFD consisted of a diet comprising 60% of calories from fat (#D12492, Research Diets, New Brunswick, NJ, USA), which included: 60% kcal from fat, 20% kcal from protein, and 20% kcal from carbohydrates, with an energy density of 5.24%. Body mass determinations were performed regularly. HFD mice received intraperitoneal injections of STZ at a dose of 40 mg/kg body weight for 5 consecutive days, with the STZ at a concentration of 1% (*w*/*v*) in 0.1 mol/L sodium citrate buffer (pH 4.5, #C1013, Solarbio), an established protocol for inducing T2DM [28]. At 1 week after STZ injections, HFD mice exhibiting two consecutive daily random blood glucose (RBG) levels exceeding 16.7 mmol/L were considered as a successful T2DM model. The second group received a normal diet (ND) and then an equivalent volume of sodium citrate buffer based on body weight to serve as a placebo control. After establishing the T2DM model, RBG was monitored regularly until the end of the experiment. Blood samples were obtained from the tails of the mice and any mouse failing to meet these glucose standards were excluded from the experiment. To evaluate the therapeutic potential of the interleukin-1 receptor antagonist (IL-1RA) for DE, recombinant mouse IL-1RA (#HY-P72566, MedChemExpress, Shanghai, China) dissolved in phosphate-buffered saline (PBS) was administered intraperitoneally at a dose of 30 mg/kg. Behavioral tests were performed 4 weeks post-injection.

### Intraperitoneal glucose tolerance test (IPGTT) and intraperitoneal insulin tolerance test (IPITT)

Mice were fasted overnight before the IPGTT and then injected intraperitoneally with glucose at a dose of 2 g/kg body weight [29]. For the IPITT, mice were fasted for 4 h and subsequently injected intraperitoneally with human insulin at a dose of 0.75 U/kg body weight. Blood glucose levels were determined at 0, 15, 30, 60, 90, and 120 min after glucose or insulin injection [29]. The blood glucose level was determined using a glucometer from tail vein blood samples.

### Hippocampal stereotaxic viral injection

Adenoviral-associated viruses (AAVs) designed to specifically overexpress or interference NLRP3 in microglia (AAV-F4/80P-NLRP3, AAV-F4/80P-shNLRP3) and their corresponding blank vectors were procured from the Shanghai GeneChem Corporation (Shanghai, China). Each of these AAVs was characterized by a titer of 1E + 12 TU/mL.

All surgical procedures were conducted under stereotaxic guidance (RWD Life Science, Shenzhen, China), with coordinates determined in relation to the bregma. Prior to surgery, animals were induced into anesthesia using isoflurane. Holes were drilled bilaterally to enable access to the hippocampus (2.00 mm posterior to the bregma, ±1.50 mm lateral to midline, 1.50 mm ventral to the dura) and 1 μL of AAV was infused at a controlled rate of 0.1 μL/min via a glass micropipette connected to a microsyringe pump (RWD Life Science). Following injections, the micropipette remained in place at the target site for an additional 10 min to allow for complete diffusion before slowly withdrawing the micropipette. Post-surgical care included thorough disinfection of the incision site and closure with sutures.

### Morris Water Maze (MWM) test

The MWM test was conducted to evaluate the spatial learning and memory capability, as described previously [30, 31]. Briefly, the circular water tank with a diameter of 150 cm and a height of 60 cm was filled with water maintained at a temperature of 22 ± 2 °C. The tank was divided into four equal quadrants with different labels pasted on the wall of each quadrant to provide a source of location for the mice. Within the tank a submerged platform with a diameter of 5 cm was positioned in the Northeast quadrant with the top surface of the platform being located 2 cm below the water surface. Swim paths were recorded using Any-maze software (Stoelting Co., Wood Dale, IL, USA). Prior to use in the experiment, mice were allowed to adapt for 1 d and those demonstrating any motor or visual impairments were eliminated from the experiment. In the learning task portion of the MWM (d 2–6), mice were trained to locate and climb onto the platform. Mice were placed in the water, always facing the wall at one of the random quadrants (except the Northeast quadrant), on each day of training. Mice were allowed 60 s to locate the platform and if located, they were rewarded with 20 s of rest on the platform while observing the location. Mice failing to locate the platform within 60 s were guided to the platform and allowed to remain on the platform for 20 s. Escaping latencies (times required to locate the platform, 60 s maximum) and the swimming trajectories were recorded. On d 7, a memory trial was conducted, during which the platform was removed. Mice were placed in the water at the Southwest quadrant and allowed to swim for a duration of 60 s. The number of target crossings, the time spend in target quadrant and the swimming trajectories were recorded.

### BV2 cell cultures and in vitro diabetes model

The BV2 cell line (mouse microglia) was purchased from the Wuhan Pricella Biotechnology Co., Ltd. (Wuhan, China) and authenticated by STR profiling. BV2 cells were cultured in Minimum Essential Medium (MEM, #C12571500BT, Gibco, Waltham, MA, USA) containing 10% fetal bovine serum (FBS, #FSP500, Excell, Shanghai, China) and 1% penicillin/streptomycin (PS, #P7630, Solarbio) in a humidified incubator (5% CO_2_ at 37 °C). The in vitro diabetes model was established by exchanging the normal concentration of glucose in the medium with that of a high glucose concentration medium (HG medium). To generate the HG medium, anhydrous glucose powder (#RDD016, Sigma-Aldrich, Darmstadt, Germany) was added to the MEM medium at the appropriate concentration. Then pH was adjusted and medium was sterilized using a 0.22 μm filter (Sevenbiotech, Beijing, China).

### Preparation of conditioned medium

To obtain conditioned medium (CM) derived from BV2 cells, cells were cultured in 6-well plates at 5 × 10^4^ cells/well and replaced with HG medium 12 h later. After cultivating BV2 cells in HG medium for 48 h, the medium was replaced with either C17.2 proliferation medium or differentiation medium. Following an additional 24 h of incubation, the two different mediums were then centrifuged at 150× *g* for 10 min at 4 °C. The CM was collected and stored at −20 °C until being thawed at 4 °C for use in the experiments.

### C17.2 cell cultures

The C17.2 cell line (mouse neuro stem cell) was purchased from Meilun Cell Center (Dalian, China) and authenticated by STR profiling. C17.2 cells were cultured in a standard humidified incubator (37 °C, 5% CO_2_). The proliferation and differentiation medium were prepared as based on a previous protocol with some minor adjustments [32]. The proliferation medium consisted of MEM medium supplemented with 10% FBS, 5% Horse Serum (HS, #16050130, Gibco) and 1% PS. The differentiation medium contained MEM medium supplemented with 2.5% FBS, 1.25% HS and 1% PS.

### Analysis of proliferation and differentiation in C17.2 cells

For analysis of cell proliferation, C17.2 cells were seeded onto poly-*L*-lysine-coated coverslips in a 24-well plate (density of 5 × 10^4^ cells/well) and subjected to the following treatment groups: 1) NC group - conventional proliferation medium, 2) CM group - conditioned medium derived from BV2 cells, 3) IL-1β group - proliferation medium containing 100 ng/mL recombinant mouse IL-1β (#HY-P7073, MedChemExpress), 4) IL-1RA group - conditioned medium containing 100 ng/mL recombinant mouse IL-RA (#HY-P72566, MedChemExpress), and 5) anti-IL-1β group - conditioned medium containing 100 ng/mL MAb anti-mouse IL-1β (#BE0246, Bio X Cell, West Lebanon, NH, USA). The dose of IL-1β selected was based on previous studies [33]. For analysis of cell differentiation, cells were induced to differentiate in the following media: 1) NC group - conventional differentiation medium, 2) CM group - conditioned medium derived from BV2 cells, 3) IL-1β group - differentiation medium containing 100 ng/mL recombinant mouse IL-1β, 4) IL-1RA group - conditioned medium containing 100 ng/mL recombinant mouse IL-RA, and 5) anti-IL-1β group - conditioned medium containing 100 ng/mL MAb anti-mouse IL-1β. On d 9, cells from the three groups were seeded onto poly-*L*-lysine-coated coverslips in 24-well plates (density of 5 × 10^4^ cells/well). Cells were fixed with 4% paraformaldehyde (PFA) for 20 min at room temperature after being in culture for 24 h, with the percent of Ki67^+^, BrdU^+^ and DCX^+^ cells then determined using immunofluorescence. For proliferation studies using the BrdU method, BrdU (10 mM, #B5002, Sigma-Aldrich) was added 2 h before fixation with cells being treated with 2 M HCl at 37 °C for 15 min prior to primary antibody incubation.

### Enzyme-Linked Immunosorbent Assay (ELISA)

BV2 cells were cultured in MEM medium supplemented with varying concentrations of glucose (5.5, 25, 50, 75, 100 or 125 mM). To control for osmotic effects, equimolar concentrations of mannitol (25, 50, 75, 100 or 125 mM) were included. At 24 h or 48 h cell culture supernatants were harvested. Samples were centrifuged at 150× *g* for 10 min at 4 °C, with supernatants collected for ELISA analysis. IL-1β (#KYY-0040M1) and IL-18 (#KYY-0169M1) were quantified using ELISA kits (Keyybio, Jinan, China) according to the manufacturer’s protocol. Detection limits were 31.25 pg/mL.

### Immunofluorescence

Immunofluorescence staining was performed as previous article [34]. Mice were anesthetized with pentobarbital sodium, followed by transcardial perfusion with 0.9% saline to remove the blood and then fixation of brain tissue with 4% PFA. After sufficient fixation, brains were removed and maintained in 4% PFA at 4 °C overnight and then dehydrated using sucrose gradients of 10%, 20%, and 30%. Brains were embedded with O.C.T. (Optimal cutting temperature) Compound (#4583, SAKURA, Japan). Finally, brains were fixed on a frozen microtome (Leica, Wetzlar, Germany) with frozen coronal hippocampal slices sectioned at 30 μm thickness. Slices were washed with 1× PBS and stored in an anti-freezing solution. After being blocked for one hour with blocking fluid containing 0.2% TritonX-100 (#BS084, Biosharp, Beijing, China), 5% goat serum (#SL038, Solarbio) or donkey serum (#SL050, Solarbio), and 25 mg/mL BSA (#A8010, Solarbio), frozen slices or fixed cells were incubated to the diluents containing primary antibodies at 4 °C overnight. Colocalization of pyroptosis-associated proteins with the microglial marker ionized calcium-binding adapter molecule 1 (IBA1, abcam, Cambridge, United Kingdom) were determined using multiple labeling immunofluorescence. To assess the transfection efficiency of AAVs, multiplex labeling immunofluorescence was performed to detect the co-localization of the Flag-tag protein with Iba1, the astrocytic marker glial fibrillary acidic protein (GFAP, Cell Signaling Technology, Danvers, MA, USA), and the neuronal marker neuronal nuclei (NeuN, Proteintech, Rosemont, IL, USA). Anti-Nestin (abcam) and Anti-SOX2 (abcam) labeled neural precursor cells, while Anti-ki67 (abcam) provided a means to identify marked cells in the proliferative phase. Anti-DCX (abcam) labeled early differentiating and immature granule cells. In C17.2 cells, Anti-BrdU (abcam) and Anti-Ki67 were used for analyzing cell proliferation, and Anti-DCX was used for analyzing cell differentiation. On the secondary day, after washing three times with PBS, slides or fixed cells were incubated with the appropriate secondary antibody for 1 h at room temperature. Information of antibodies used is available in Supplementary Table S1. Finally, the Mounting Medium with DAPI (#ab104139, abcam) was used for nuclear staining. The entire process was performed in the dark and slices or fixed cells were sealed as soon as possible. Images were captured under an inverted confocal microscope (#LSM800, Zeiss, Oberkochen, Germany) and processed by Zeiss ZEN software. Cell counting was performed in a blinded manner using ImageJ Fiji software (https://imagej.net/software/fiji/downloads). Only cells exhibiting specific fluorescent signals, possessing intact cellular morphology, and located within the hippocampal dentate gyrus were included in the final counts. Three brain sections were randomly selected for each animal. Using a 10× objective and the DAPI channel, the overall contour of the DG region was defined based on the densely packed nuclear layer of the granule cells. Within this contour, five non-overlapping fields of view were randomly selected for imaging. Higher-magnification images were then acquired for each field. All analyses were performed blinded. The Manders’ Colocalization Coefficients (MCC) for Iba1 with NLRP3, caspase-1, GSDMD, or IL-1β were quantified using the Colocalization Finder plugin in ImageJ Fiji, and the mean MCC from the five fields per section was calculated.

### Real-time quantitative PCR (RT-qPCR)

RNA from hippocampal tissues was extracted using the EZ-press RNA Purification Kit (#RN001, EZBioscience, Roseville, MN, USA), while cDNA synthesis was evaluted with use of Evo M-MLV reverse transcription Kit II (#AG11705, Accurate Biology, Changsha, China) according to the operating instructions. RT-qPCR was performed using the ChamQ SYBR qPCR Master Mix (#Q111-02, Vazyme, Nanjing, China) and was detected with use of the QuantStudio™ 5 Real-time fluorescence quantitative PCR system (ThermoFisher Scientific, Waltham, MA, USA). The Actin gene served as the housekeeping internal control. Gene expression was quantified as relative expression levels using the 2^−ΔΔCt^ method. Primer sequences for the genes are listed in Supplementary Table S2.

### Western blot

Hippocampal tissues or BV2 cells were lysed on ice for 30 min using RIPA buffer (#P0013B, Beyotime, Shanghai, China) containing a protease inhibitor cocktail and phenylmethylsulfonyl fluoride (PMSF, #st506, Beyotime). Following lysis, the samples were sonicated and centrifuged at 12,000× *g* for 15–20 min at 4 °C. The supernatant was collected, and protein concentrations were measured with a BCA Protein Assay Kit (#PC0020, Solarbio). Samples were mixed with 5× Loading Buffer (#P0295, Beyotime) and heated at 100 °C for 10 min. The mixtures were added to SDS-PAGE gels for electrophoresis and subsequently transferred onto PVDF membranes (Merck Millipore, Darmstadt, Germany). The PVDF membranes were blocked with 5% skim milk for 1 h at room temperature and incubated overnight at 4 °C with diluted primary antibodies (Rabbit anti-NLRP3, IgG, #ab263899, abcam; Rabbit anti-caspase1, IgG, #22915-1-AP, proteintech; Rabbit anti-GSDMD, IgG, #ab209845, abcam; Rabbit anti-IL-1β, IgG, #A16288, ABclonal; Rabbit anti-β-actin, IgG, #AC026, ABclonal; Rabbit anti-β-tubulin, IgG, #A12289, ABclonal). On the following day, after washing three times with TBST, membranes were incubated with secondary antibodies conjugated with horseradish peroxidase (goat anti-rabbit IgG, 1:5000, #ZB2301, ZSJQ-Bio, Beijing, China) for 1 h at room temperature. An enhanced chemiluminescence detection kit (#WBULS0500, Merck Millipore) was used to assess the images as detected under a chemiluminescence imager (#4800, Tanon, Shanghai, China). The obtained band images were imported into ImageJ for analysis.

### Statistical analysis

Behavioral tests and statistical analysis were performed according to double-blind experiment conditions. All data were collected and analyzed as means ± SEMs. Statistical significance of differences between two groups were evaluated using a two-tailed independent-samples *t* test while those with greater than two groups with a one-way analysis of variance (ANOVA) followed by the Tukey or Dunnett test for *post-hoc* pairwise comparisons. For the time course studies, the level of significance was determined using the two-way ANOVA followed by the Tukey or Holm–Šidak multiple comparisons test. All statistical analyses were performed using the GraphPad Prism 9.5.1 software. Only *P* < 0.05 was required for results to be considered statistically significant.

## Results

### Cognitive ability is impaired in DE mice

**Fig. 1:**
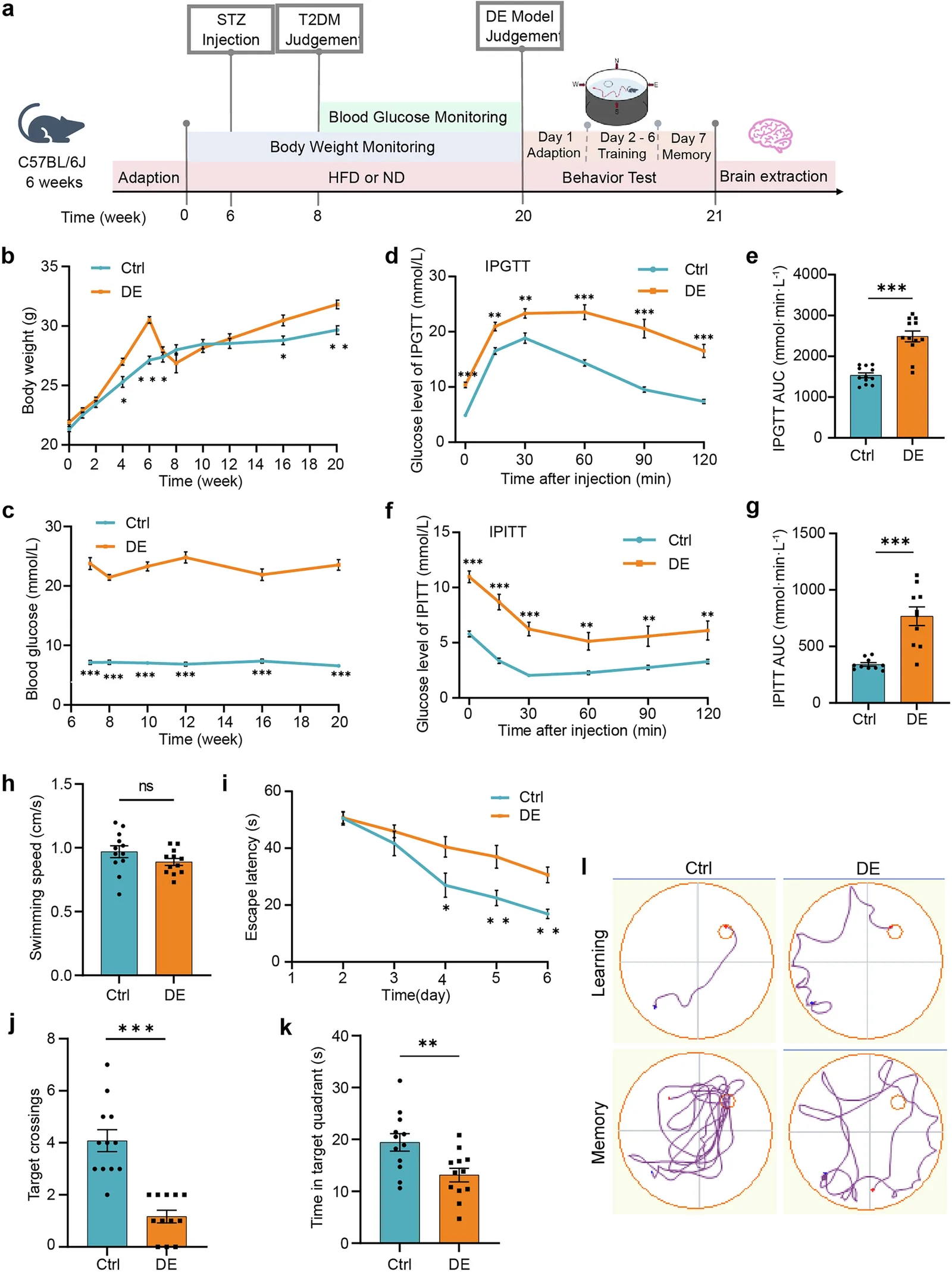
DE mice show reduced spatial learning and memory function. **a** Experimental paradigm for the animal model of diabetic encephalopathy. **b** Body weight and **c** random blood glucose level in Ctrl and DE group (*n* = 12). **d** The glucose level at 0, 15, 30, 60, 90 and 120 min during the IPGTT in Ctrl and DE group after intraperitoneal administration of 2 g/kg glucose (*n* = 11). **e** The area under the curves (AUC) was calculated for glucose of IPGTT (*n* = 11). **f** The glucose level at 0, 15, 30, 60, 90 and 120 min during the IPITT in Ctrl and DE group after intraperitoneal administration of 0.75 U/kg insulin (*n* = 10). **g** The AUC was calculated for glucose of IPITT (*n* = 10). **h** Swimming speed in the MWM test (*n* = 12). **i** Escape latency during the MWM training period (*n* = 12). **j** Number of target crossings in the probe trail period (*n* = 12). **k** The time spent in target quadrant in the probe trail period (*n* = 12). **l** Representative motor trajectories on the learning period and the probe trail period in Ctrl and DE mice. The difference was analyzed using unpaired two-tailed *t* test or two-way ANOVA. All data are shown as means ± SEMs. ^ns^ *P* > 0.05, *P < 0.05, **P < 0.01, and ***P < 0.001 Ctrl vs. DE. Ctrl Control group. DE Diabetic Encephalopathy group.

We established a T2DM mouse model using HFD combined with intraperitoneal injection of STZ, and assessed the spatial learning and memory functions of the mice using the MWM test at 12 weeks after a successful establishment of the T2DM mouse model. Figure 1a contains a schematic illustration of this experimental chronology. Compared to Ctrl mice, DE mice exhibited an increase in body weight, following the administration of HFD diet (Fig. 1b). Additionally, DE mice displayed higher levels of random blood glucose (RBG) after the injection of STZ (Fig. 1c). DE mice exhibited impaired glucose tolerance, which was manifested as increased blood glucose value at each detection time point (Fig. 1d) and increased glucose-time AUC (Fig. 1e) in IPGTT. In addition, DE mice exhibited reduced insulin sensitivity, which was manifested as increased blood glucose value at each detection time point (Fig. 1f) and increased glucose-time AUC (Fig. 1g) in IPITT. There were no significant differences in swimming speeds between the Ctrl and DE groups (Fig. 1h), indicating that the alterations in learning and memory assessments in the MWM test were not affected by differences in swimming ability. From d 4 of the MWM test, escape latencies of DE mice were markedly prolonged (Fig. 1i, l). During the probe trial period, DE mice made fewer platform crossings (Fig. 1j, l) and spend less time in the target quadrant (Fig. 1k). Collectively, these findings suggest that DE mice showed impaired spatial learning and memory functions.

### Neurogenesis is reduced in the hippocampal DG region of DE mice

**Fig. 2:**
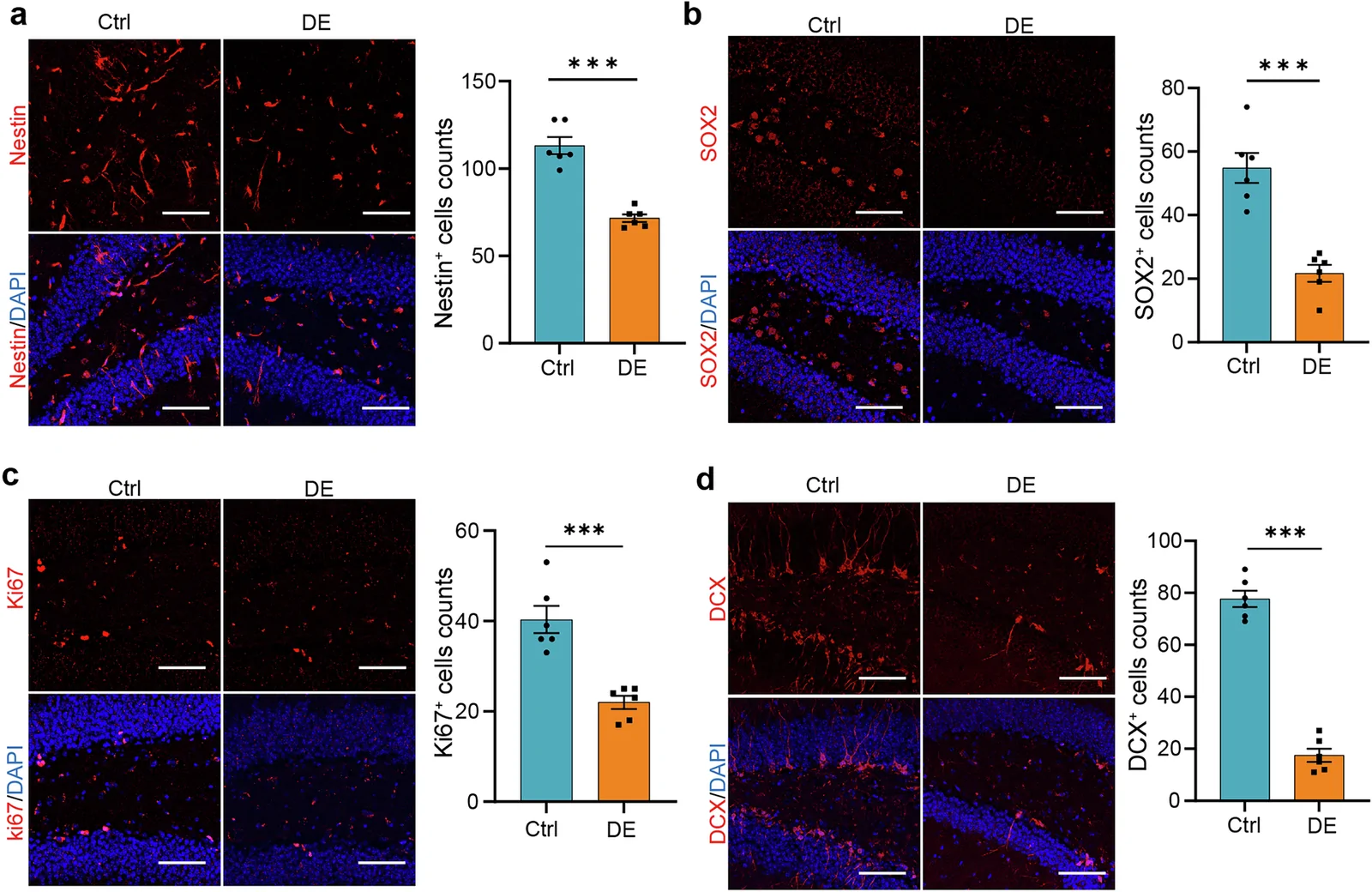
AHN is reduced in DG regions of DE mice. **a** Representative confocal microscopic images of immunostainings and quantification for Nestin^+^ cells in DG regions of the hippocampus (*n* = 6). Scale bars, 100 μm. **b** Representative confocal microscopic images of immunostainings and quantification for SOX2^+^ cells in DG regions of the hippocampus (*n* = 6). Scale bars, 100 μm. **c** Representative confocal microscopic images of immunostainings and quantification for Ki67^+^ cells in DG regions of the hippocampus (*n* = 6). Scale bars, 100 μm. **d** Representative confocal microscopic images of immunostainings and quantification for DCX^+^ cells in DG regions of the hippocampus (*n* = 6). Scale bars, 100 μm. The difference was analyzed using unpaired two-tailed *t* test. All data are shown as means ± SEMs. ***P < 0.001, Ctrl vs. DE. Ctrl, Control group. DE Diabetic Encephalopathy group.

Given that hippocampal-related behavioral deficits are often associated with AHN deficiencies, we proceeded to investigate the neurogenic activity in hippocampal DG region of DE mice. Neural precursor cells can be labeled by Nestin and SOX2, and proliferating cells can be labeled by Ki67. Results revealed a significant reduction in the number of Nestin^+^ (Fig. 2a), SOX2^+^ (Fig. 2b) and Ki67^+^ cells (Fig. 2c) in the hippocampal DG region of the DE group versus Ctrl group. To evaluate NSCs in the proliferative phase within the DG, brain sections were collected following intraperitoneal BrdU injection in mice. Both BrdU⁺/Nestin⁺ (Supplementary Fig. S1a) and BrdU⁺/SOX2⁺ cell (Supplementary Fig. S1b) populations were reduced in DE mice, indicating a significant suppression of NSCs proliferation. DCX support the existence of early differentiated and immature granule cells, and immunofluorescence analysis revealed a significant decrease in DCX^+^ cells (Fig. 2d) and BrdU^+^/DCX^+^ cells (Supplementary Fig. S1c) in DE mice, indicating that the differentiation of NSCs in the DG region was inhibited. In summary, the AHN in the hippocampal DG region of DE mice was impaired, as evidenced by a decrease in the number of neuro precursor cells and the proliferation and differentiation of NSCs.

### Microglial pyroptosis is increased within the hippocampus of DE mice

**Fig. 3:**
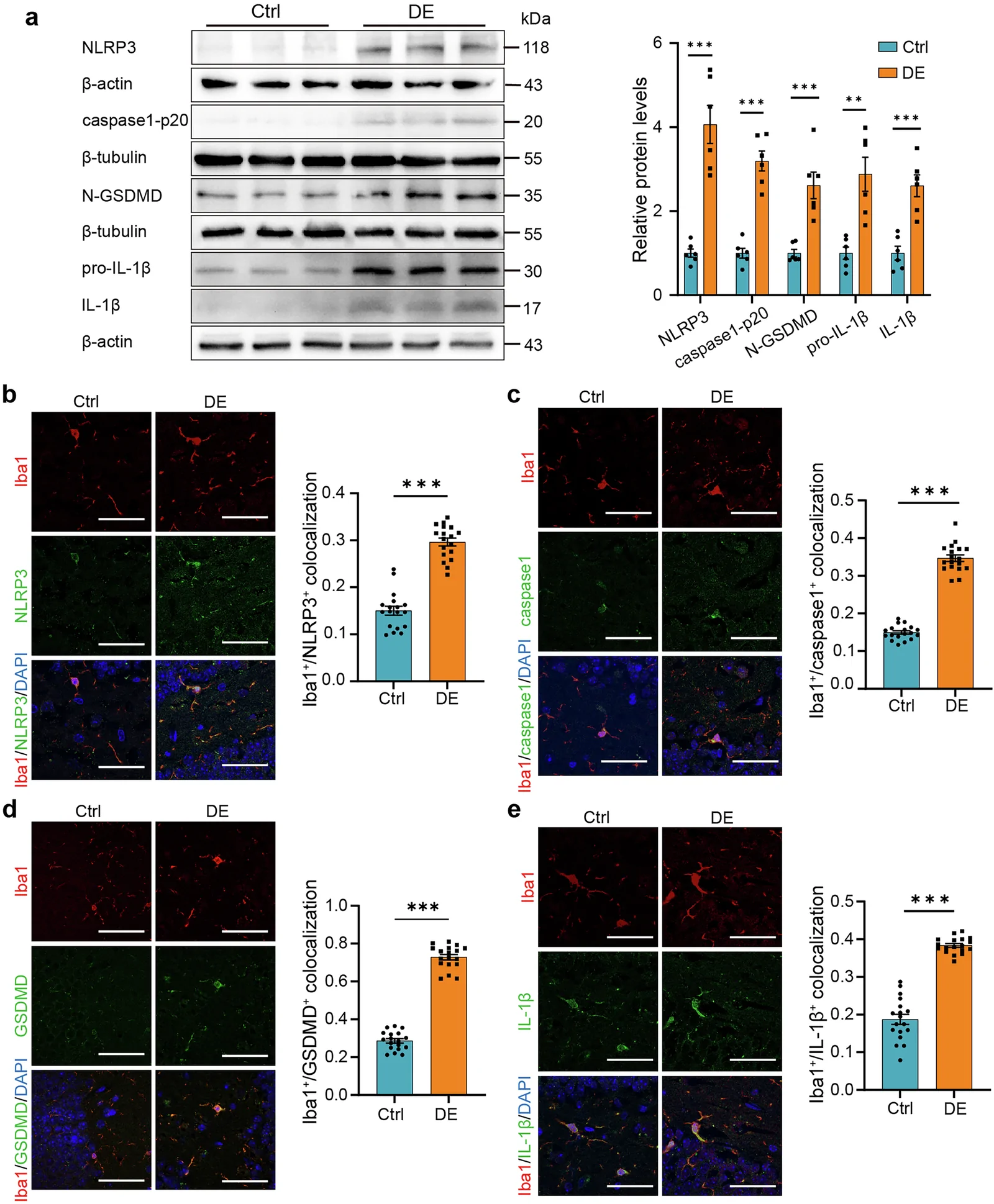
Exacerbated microglial pyroptosis in hippocampal of DE mice. **a** Representative Western blot images and relative protein levels of NLRP3, caspase-1-p20, N-GSDMD and IL-1β in the hippocampus of Ctrl and DE mice (*n* = 6). **b** Representative confocal microscopic images and colocalization analysis of Iba1 and NLRP3 in DG regions of Ctrl and DE mice (*n* = 18 images per group). Scale bars, 50 μm. **c** Representative confocal microscopic images and colocalization analysis of Iba1 and caspase-1 in DG regions of Ctrl and DE mice (*n* = 18 images per group). Scale bars, 50 μm. **d** Representative confocal microscopic images and colocalization analysis of Iba1 and GSDMD in DG regions of Ctrl and DE mice (*n* = 18 images per group). Scale bars, 50 μm. **e** Representative confocal microscopic images and colocalization analysis of Iba1 and IL-1β in DG regions of Ctrl and DE mice (*n* = 18 images per group). Scale bars, 50 μm. All IF images are representative of six independent biological replicates, with three technical images analyzed per replicate. The difference was analyzed using unpaired two-tailed *t* test. All data are shown as means ± SEMs. **P < 0.01, and ***P < 0.001, Ctrl vs. DE. Ctrl Control group, DE Diabetic Encephalopathy group.

It is well established that microglia are the primary immune effector cells in the central nervous system. As important components of the neurogenic niches, microglia participate in the regulation of AHN. Pyroptosis of microglia, mediated by the NLRP3 inflammasome, leads to the release of pro-inflammatory cytokines, which can cause AHN disorder. We next investigated whether DE mice exhibit NLRP3-dependent microglial pyroptosis. Western blot analysis revealed an upregulation of NLRP3, caspase-1-p20, N-GSDMD and IL-1β protein levels in the hippocampal tissue of DE mice (Fig. 3a). The main executive proteins and the key pro-inflammatory cytokine were increased, indicating that NLRP3-dependent pyroptosis in the hippocampal of DE mice were aggravated. With use of immunofluorescence, we found a significant increase in the colocalization of Iba1 with NLRP3 (Fig. 3b), caspase-1 (Fig. 3c), GSDMD (Fig. 3d) and IL-1β (Fig. 3e) within the hippocampal DG of DE mice, indicating that the increased expression of pyroptosis related proteins mainly occurred in microglia. These findings suggest that NLRP3 inflammasome formation in microglia leads to increased activation of caspase-1, GSDMD and IL-1β and aggravation of pyroptosis in the hippocampal DG region of DE mice.

### The expression of NLRP3 in microglia affects the cognitive function of mice

**Fig. 4:**
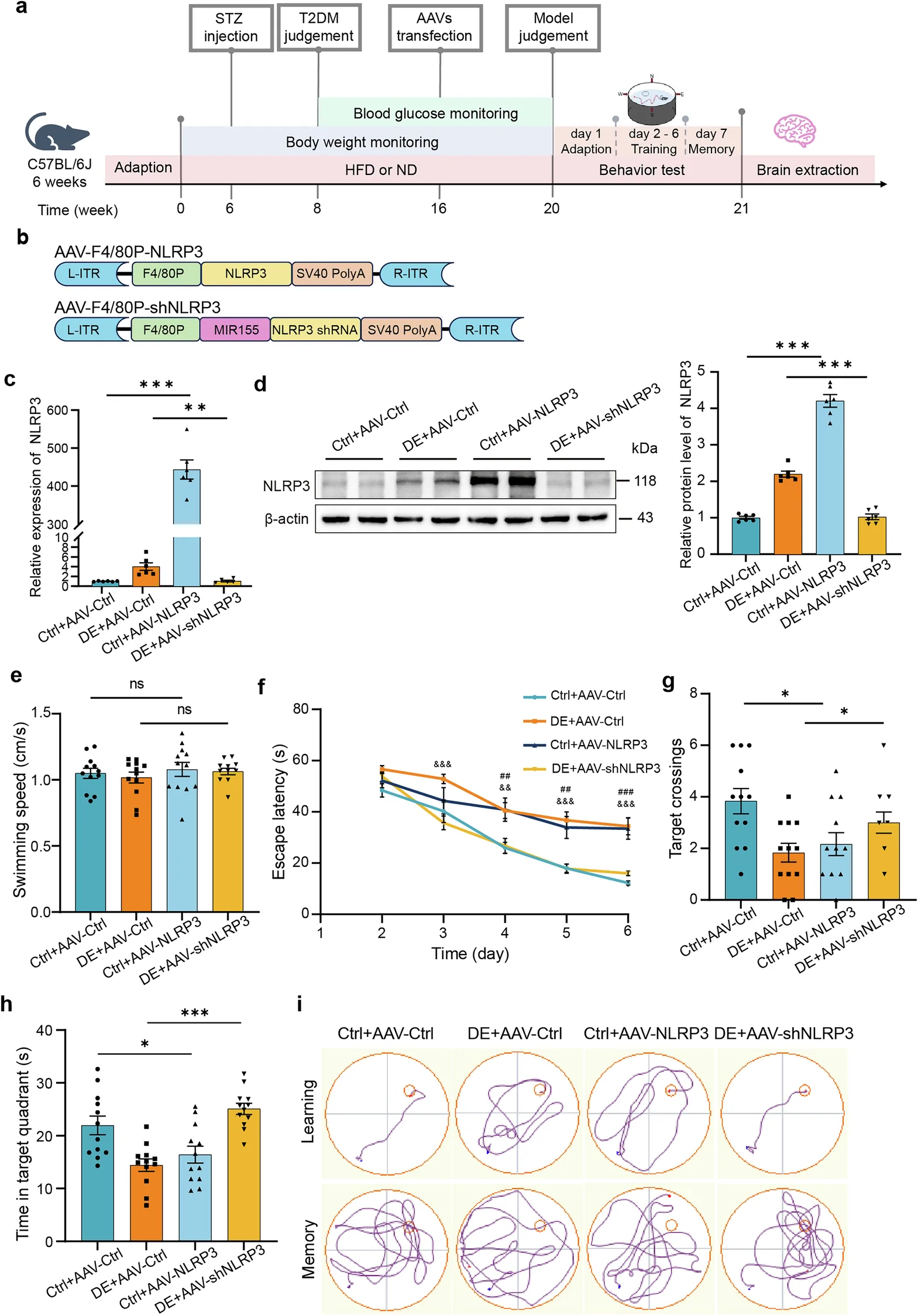
Expression of NLRP3 in microglia affects cognitive function in mice. **a** Experimental paradigm for AAVs injection and the MWM test in mice. **b** Schematic of element sequence of AAVs regulating NLRP3 expression in microglial. **c** Relative mRNA expression levels of NLRP3 in the hippocampus after AAVs injection as determined using RT-qPCR (*n* = 6). **d** Representative Western blot images and relative protein levels of NLRP3 in the hippocampus after AAVs injection (*n* = 6). **e** Swimming speed of the four groups in the MWM test (*n* = 12). **f** Escape latency during the MWM training period (*n* = 12). The difference was analyzed using two-way ANOVA. All data represent means ± SEMs. ^##^*P* < 0.01, ^###^*P* < 0.001, Ctrl + AAV-Ctrl group vs. Ctrl + AAV-NLRP3 group; ^& &^*P* < 0.01, ^& & &^*P* < 0.001, DE + AAV-Ctrl group vs. DE + AAV-shNLRP3 group. **g** Number of target crossings in the MWM probe trail period (*n* = 12). **h** The time spent in target quadrant in the probe trail period (*n* = 12). (**i**) Representative motor trajectories on the MWM training period and the probe trail period. Except for escape latency, all other differences were analyzed using one-way ANOVA. All data are shown as means ± SEMs. ^ns^ *P* > 0.05, *P < 0.05, **P < 0.01, and ***P < 0.001, Ctrl + AAV-Ctrl group vs. Ctrl + AAV-NLRP3 group, DE + AAV-Ctrl group vs. DE + AAV-shNLRP3 group.

To determine whether the expression of NLRP3 in microglia is a critical risk factor for cognitive decline in DE mice and to explore the regulatory mechanisms, we constructed AAV-NLRP3 and AAV-shNLRP3 with specific promoter F4/80 P to specifically modulate NLRP3 expression in microglia. We bilaterally injected AAV-F4/80-NLRP3 and corresponding blank vectors AAV-F4/80-Ctrl into the hippocampus of Ctrl mice (Ctrl + AAV-NLRP3 group and Ctrl + AAV-Ctrl group), and similarly injected AAV-F4/80-shNLRP3 and corresponding blank vectors AAV-F4/80-Ctrl into the hippocampus of DE mice (DE + AAV-shNLRP3 group and DE + AAV-Ctrl group). Figure 4a contains a schematic illustration of this experimental chronology and the viral vector sequences used are shown in Fig. 4b. Immunofluorescence analysis revealed colocalization of the AAV-derived Flag tag with Iba1 (Supplementary Fig. S2a). While low-level Flag expression was present in astrocytes (GFAP^+^) (Supplementary Fig. S2b), its colocalization with GFAP was significantly lower than with Iba1 (Supplementary Fig. S2c). Neurons (NeuN^+^) (Supplementary Fig. S2d) showed no detectable Flag signal. Together, these data confirm the high microglial selectivity of the F4/80-AAV in our experimental paradigm. After injection with AAV-F4/80-NLRP3, there was a remarkable upregulation of NLRP3 mRNA levels (Fig. 4c) and protein expression (Fig. 4d) in the hippocampal tissue of Ctrl mice. After injection with AAV-F4/80-shNLRP3, there was a significant downregulation of NLRP3 mRNA levels (Fig. 4c) and protein expression (Fig. 4d) in the hippocampal tissue of DE mice, confirming the efficacy of the viral infection. Four weeks after viral injection, the MWM test was conducted. There were no statistically significant differences in the swimming speeds of mice across the four groups (Fig. 4e). Compared to the Ctrl + AAV-Ctrl group, the Ctrl + AAV-NLRP3 group exhibited significantly prolonged escape latencies (Fig. 4f, i) during the learning period. In the probe trial period, this group exhibited marked fewer platform crossings (Fig. 4g, i) and less time spend in target quadrant (Fig. 4h). In contrast, the DE + AAV-shNLRP3 group showed significant reductions in escape latencies (Fig. 4f, i), an increase in platform crossings (Fig. 4g, i) and a longer duration in target quadrant (Fig. 4h) compared to the DE + AAV-Ctrl group. These results indicated that upregulation of NLRP3 expression in microglia impairs spatial learning and memory function in Ctrl mice, while downregulation of NLRP3 in microglia ameliorates cognitive dysfunction in DE mice.

### The expression of NLRP3 in microglia affects AHN in mice

**Fig. 5:**
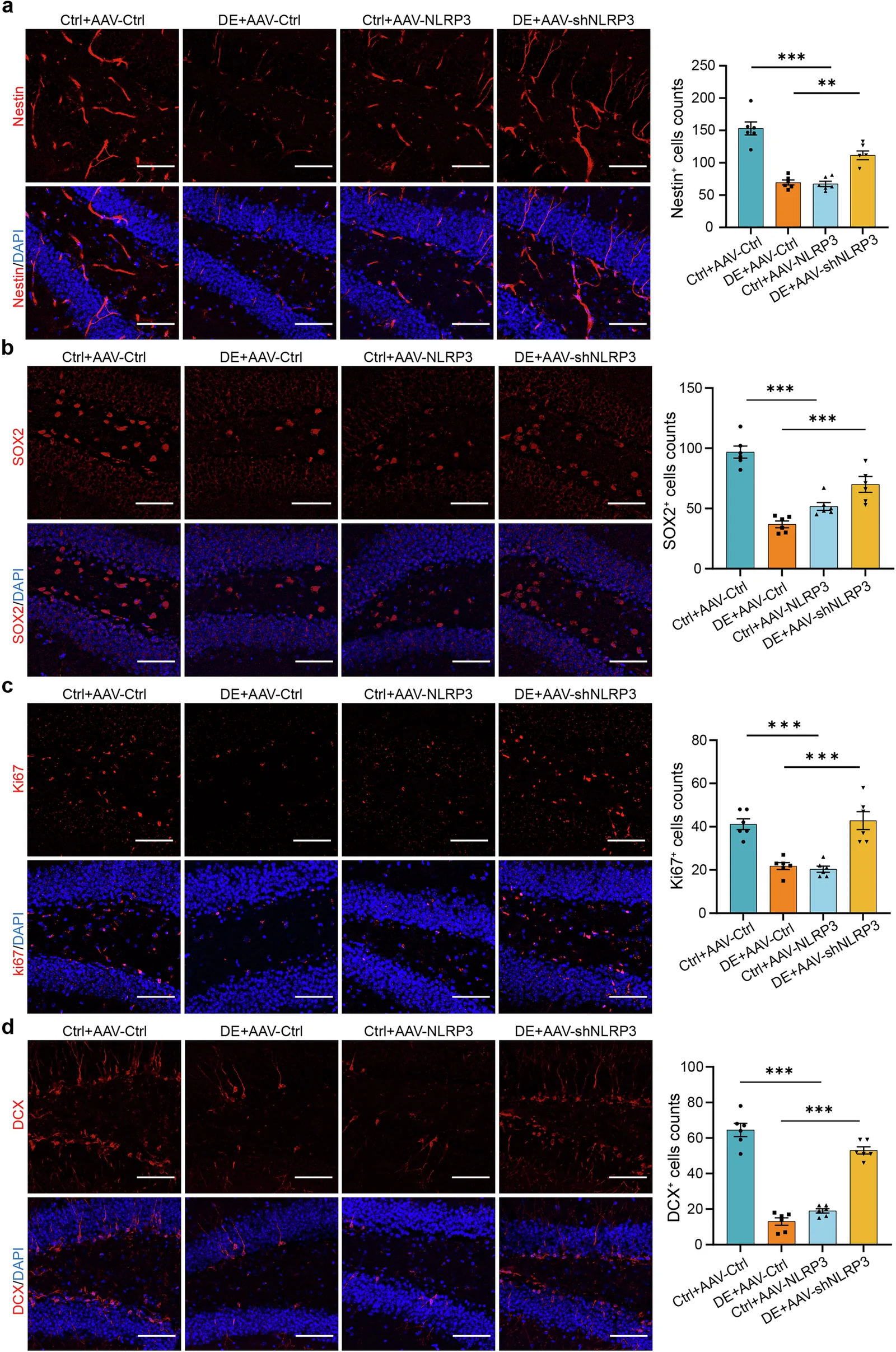
The expression of NLRP3 in microglia is associated with AHN in DG regions. **a** Representative confocal microscopic images of immunostainings and quantification for Nestin^+^ cells in DG regions of the hippocampus (*n* = 6). Scale bars, 100 μm. **b** Representative confocal microscopic images of immunostainings and quantification for SOX2^+^ cells in DG regions of the hippocampus (*n* = 6). Scale bars, 100 μm. **c** Representative confocal microscopic images of immunostainings and quantification for Ki67^+^ cells in DG regions of the hippocampus (*n* = 6). Scale bars, 100 μm. **d** Representative confocal microscopic images of immunostainings and quantification for DCX^+^ cells in DG regions of the hippocampus (*n* = 6). Scale bars, 100 μm. The difference was analyzed using one-way ANOVA. All data are shown as means ± SEMs. **P < 0.01 and ***P < 0.001, Ctrl + AAV-Ctrl group vs. Ctrl + AAV-NLRP3 group, DE + AAV-Ctrl group vs. DE + AAV-shNLRP3 group.

To investigate the impact of NLRP3 expression in microglia on neurogenesis, we examined AHN in the DG region of hippocampus. Our immunofluorescent assay results revealed that, compared to the Ctrl + AAV-Ctrl mice, the number of Nestin^+^ (Fig. 5a), SOX2^+^ (Fig. 5b), Ki67^+^ (Fig. 5c), and DCX^+^ cells (Fig. 5d) in the hippocampal DG of the Ctrl + AAV-NLRP3 mice were significantly reduced. In contrast, the number of Nestin^+^ (Fig. 5a), SOX2^+^ (Fig. 5b), Ki67^+^ (Fig. 5c), and DCX^+^ cells (Fig. 5d) in the hippocampal DG of the DE+shNLRP3 mice were significantly increased compared to the DE + AAV-Ctrl mice. Taken together, these findings infer that the expression of NLRP3 in microglia can regulate AHN. Upregulation of NLRP3 expression within microglia leads to the impaired AHN in Ctrl mice, whereas downregulation of NLRP3 expression within microglia mitigates the impairment of AHN in DE mice.

### NLRP3 regulates microglial pyroptosis in the hippocampus of DE mice

**Fig. 6:**
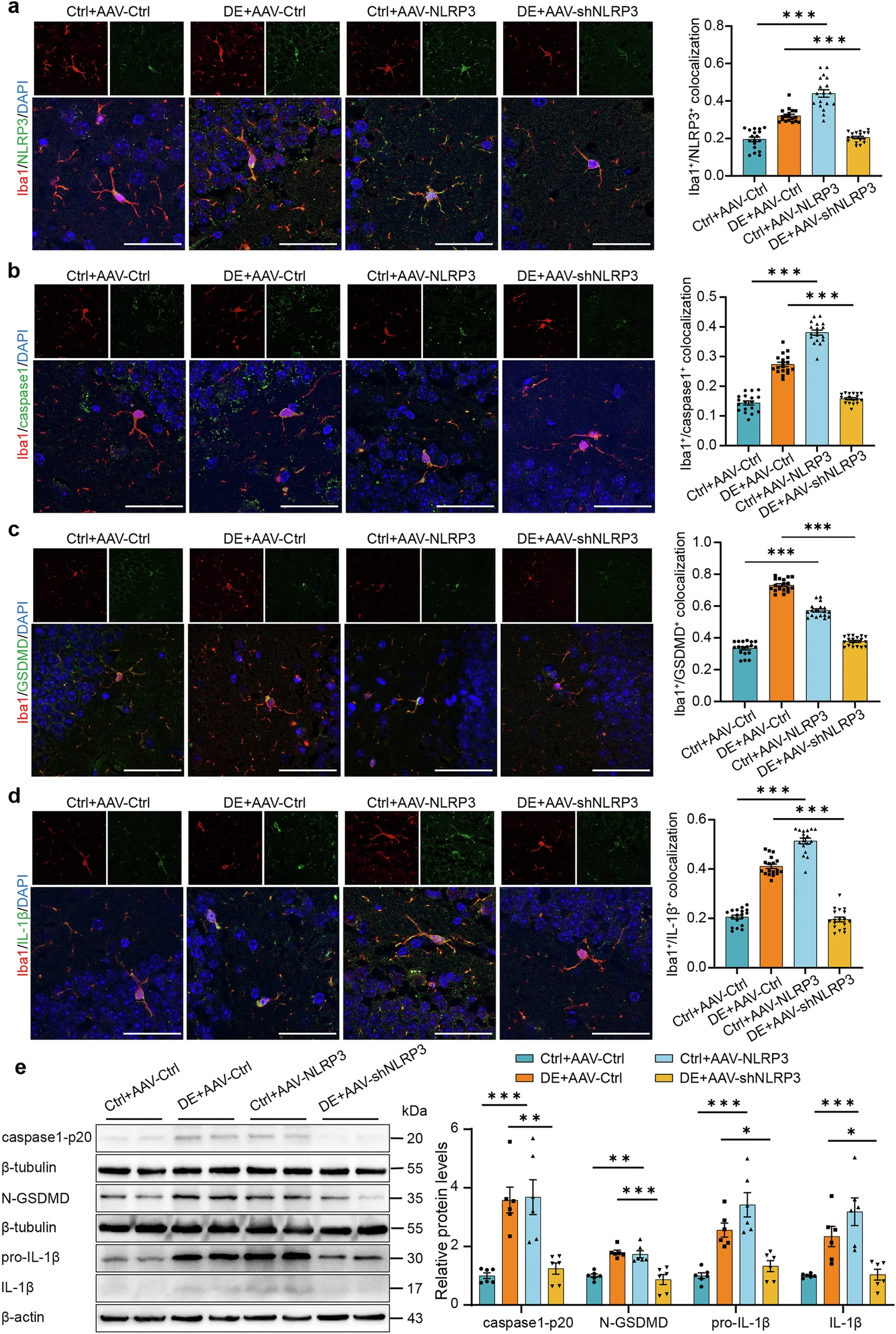
NLRP3 regulates microglial pyroptosis in the hippocampus. **a** Representative confocal microscopic images and colocalization analysis of Iba1 and NLRP3 in DG regions (*n* = 18 images per group). Scale bars, 50 μm. **b** Representative confocal microscopic images and colocalization analysis of Iba1 and caspase-1 in DG regions (*n* = 18 images per group). Scale bars, 50 μm. **c** Representative confocal microscopic images and colocalization analysis of Iba1 and GSDMD in DG regions (*n* = 18 images per group). Scale bars, 50 μm. **d** Representative confocal microscopic images and colocalization analysis of Iba1 and IL-1β in DG regions (*n* = 18 images per group). Scale bars, 50 μm. **e** Representative Western blot images and relative protein levels of caspase-1-p20, N-GSDMD and IL-1β in the hippocampus of the four groups (*n* = 6). All IF images are representative of six independent biological replicates, with three technical images analyzed per replicate. The difference was analyzed using one-way ANOVA. All data are shown as means ± SEMs. *P < 0.05, **P < 0.01, and ***P < 0.001, Ctrl + AAV-Ctrl group vs. Ctrl + AAV-NLRP3 group, DE + AAV-Ctrl group vs. DE + AAV-shNLRP3 group.

To further elucidate the potential mechanisms underlying NLRP3-induced impairment of AHN, we examined the microglial pyroptosis in hippocampus. Our immunofluorescent analysis revealed that the colocalization of Iba1 with NLRP3 (Fig. 6a), caspase-1 (Fig. 6b), GSDMD (Fig. 6c), and IL-1β (Fig. 6d) in the hippocampus was significantly enhanced in the Ctrl + AAV-NLRP3 group compared to the Ctrl + AAV-Ctrl group, which reflected a deterioration of pyroptosis in microglia. Conversely, decreased levels of NLRP3 attenuated pyroptosis observed within the microglia of DE mice, immunofluorescent analysis revealed that the colocalization of Iba1 with NLRP3 (Fig. 6a), caspase-1 (Fig. 6b), GSDMD (Fig. 6c), and IL-1β (Fig. 6d) in the hippocampus was significantly decreased in the DE + AAV-shNLRP3 groups. Western blot results of caspase-1-p20, N-GSDMD and IL-1β further verified that pyroptosis was regulated by NLRP3, as shown in Fig. 6e. Specifically, compared to the Ctrl + AAV-Ctrl group, expression levels of caspase-1-p20, N-GSDMD and IL-1β were significantly elevated in the Ctrl + AAV-NLRP3 groups. In the DE + AAV-shNLRP3 group, the expression levels of caspase-1-p20, N-GSDMD and IL-1β were reversed with the knockdown of NLRP3 (Fig. 6e). These results suggested that NLRP3 in hippocampal microglia might be involved in the pathogenesis of DE by regulating microglial pyroptosis and neuroinflammation.

### NLRP3 expression and IL-1β secretion are increased in BV2 cells cultured in high glucose

**Fig. 7:**
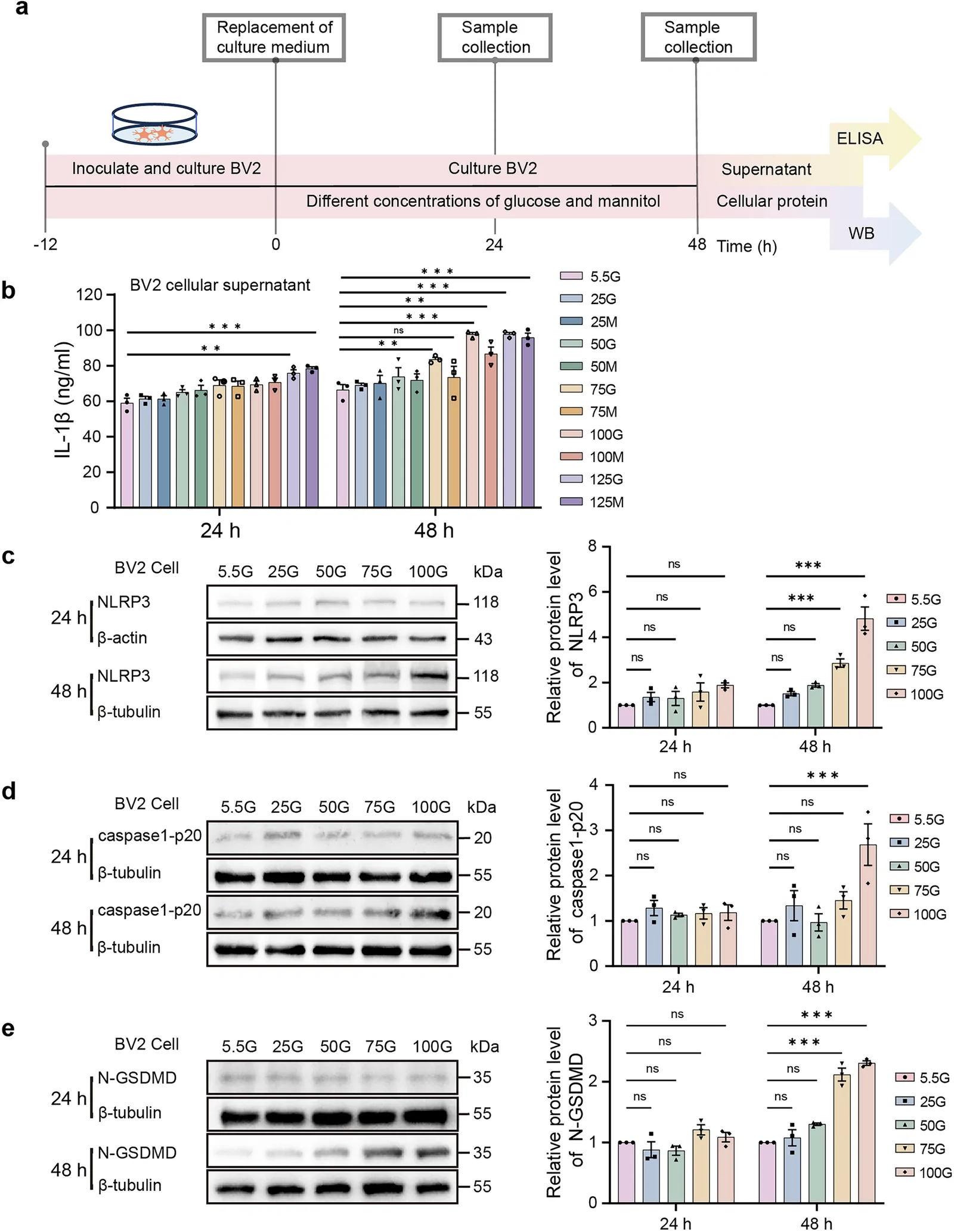
Microglial pyroptosis is increased in an in vitro diabetic model. **a** Schematic diagram of pyroptosis induction in microglia (BV2 cells) by high-glucose. **b** The levels of IL-1β in the supernatant of BV2 cells cultured with different concentrations of glucose or mannitol for 24 or 48 h, as measured by ELISA (*n* = 3 independent experiments). **c** Representative Western blot images and relative protein levels of NLRP3 within BV2 cells cultured with different concentrations of glucose for 24 or 48 h (*n* = 3 independent experiments). **d** Representative Western blot images and relative protein levels of caspase-1-p20 within BV2 cells cultured with different concentrations of glucose for 24 or 48 h (*n* = 3 independent experiments). **e** Representative Western blot images and relative protein levels of N-GSDMD within BV2 cells cultured with different concentrations of glucose for 24 or 48 h (*n* = 3 independent experiments). The difference was analyzed using two-way ANOVA. All data are shown as means ± SEMs. **P < 0.01, ***P < 0.001, 5.5 G group vs. other groups. G, Glucose. M, Mannitol. For example, 5.5 G, 5.5 mM Glucose; 25 M, 25 mM Mannitol.

The general design of this experiment is illustrated in the schematic diagram of Fig. 7a. ELISA was used to measure the levels of IL-1β or IL-18 in cell supernatants at specific time points (24 h, 48 h). As shown in Fig. 7b, the IL-1β content in the supernatant was increased along with the increase of glucose concentration and culture time and we chose the 75 mM at 48 h as the conditions for testing the effects of high glucose (HG) levels, results of the statistical analysis were shown in figure. The level of IL-18 in the supernatant also increased with higher glucose concentrations and longer incubation periods (Supplementary Fig. S3). As shown in Fig. 7c–e, Western blot analysis revealed no statistically significant differences in the expression of NLRP3, caspase-1-p20, and N-GSDMD proteins among BV2 cells cultured for 24 h in media with different concentrations of glucose. After culturing BV2 cells in media with 75 mM and 100 mM glucose for 48 h, expression levels of NLRP3, caspase-1-p20, and N-GSDMD were all significantly increased, results of the statistical analysis were shown in figures. Combining these results, it is evident that as glucose concentration increases and culture time extends, NLRP3-dependent pyroptosis and the release of IL-1β of BV2 cells increase.

### IL-1β released from microglia mediates AHN impairment

To confirm the increased microglial pyroptosis leading to AHN impairment observed in animal models and to further explore the connection between these phenomena, DE mice were administered IL-1RA via intraperitoneal injection. No significant difference in swimming speed was detected between the two groups in the MWM test (Supplementary Fig. S4a). During the learning period, the IL-1RA-treated group exhibited a reduction in escape latency compared to the DE group (Supplementary Fig. S4b, e). During the probe trial period, the IL-1RA-treated group showed an increase in the number of platform crossings (Supplementary Fig. S4c, e) and spent more time in the target quadrant (Supplementary Fig. S4d). These behavioral findings suggest that the treatment with IL-1RA ameliorates cognitive impairment in DE mice. Immunofluorescence analysis showed that compared to the DE group, the number of Nestin^+^ cells, SOX2^+^ cells, Ki67^+^ cells, and DCX^+^ cells in the DG of the IL-1RA-treated group increased (Supplementary Fig. S5a–d), indicating that IL-1RA treatment alleviates AHN impairment.

**Fig. 8:**
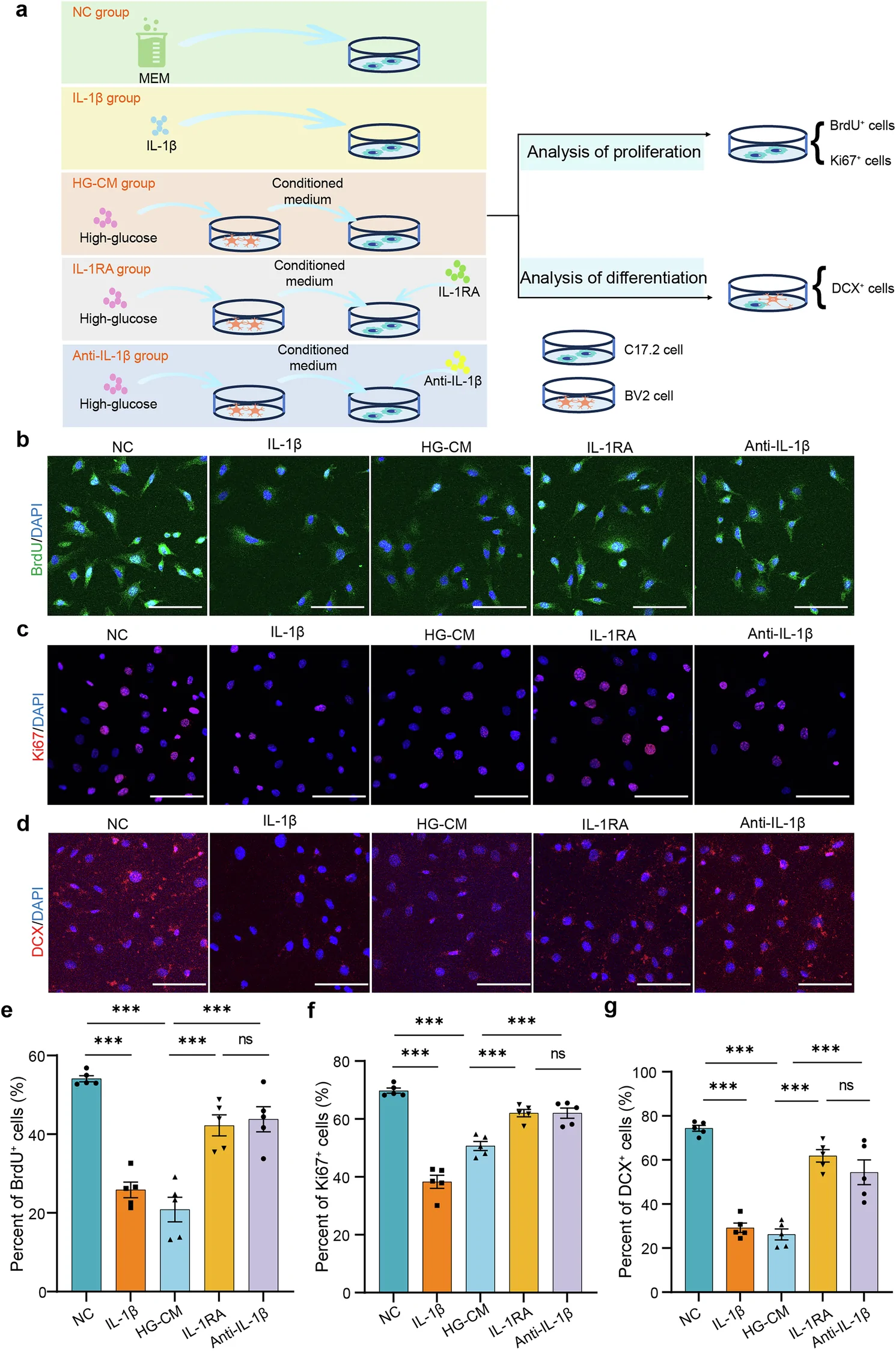
IL-1β affects the proliferation and differentiation of C17.2 cells. **a** Experimental paradigms for the in vitro experiment. Proliferation (**b**, **c**) and differentiation (**d**) of C17.2 cells assessed by immunofluorescence in vitro. **b**, **e** Representative confocal microscopic images and quantification for BrdU^+^ cells (*n* = 5). Scale bars, 100 μm. **c**, **f** Representative confocal microscopic images and quantification for Ki67^+^ cells (*n* = 5). Scale bars, 100 μm. **d**, **g** Representative confocal microscopic images and quantification for DCX^+^ cells (*n* = 5). Scale bars, 100 μm. All IF images are representative of five independent biological replicates, with data from three technical images per replicate presented as the mean. The difference was analyzed using one-way ANOVA. All data are shown as means ± SEMs. ***P < 0.001, NC group vs. IL-1β group, NC group vs. HG-CM group, HG-CM group vs. IL-1RA group. IL-1β recombinant mouse IL-1β group, HG-CM conditioned medium derived from high-glucose-treated BV2 cells group, IL-1RA the group with the addition of recombinant mouse IL-1RA to the conditioned medium, anti-IL-1β the group with the addition of MAb anti-mouse-IL-1β to the conditioned medium.

In addition, we constructed an in vitro model using the BV2 and C17.2 cell lines. The overall design of this experimental procedure is depicted in Fig. 8a. As established in previous studies [32], the proliferation medium and differentiation medium respectively promote cell proliferation and differentiation. Randomly selected microscopic fields showed that cells in the proliferation medium proliferated significantly, while those in the differentiation medium exhibited typical morphological changes, including smaller cell bodies and extended neurites, contrasting with the irregular shape of undifferentiated cells (Supplementary Fig. S6a, b). Confocal imaging showed a significant increase in BrdU⁺ and Ki67⁺ cell percentages following 3-day culture in proliferation medium (Supplementary Fig. S6c, d). Differentiation medium, in contrast, demonstrated a marked rise in DCX⁺ cells, with proliferation medium showing no significant differentiation (Supplementary Fig. S6e). The percentages of BrdU^+^ and Ki67^+^ cells can reflect the proliferation rate of C17.2 cells, while the percentage of DCX^+^ cells can indicate the differentiation rate. Immunofluorescence analysis revealed that compared to the NC group, the percentages of BrdU^+^, Ki67^+^ cells, and DCX^+^ cells in the IL-1β group and HG-CM group were significantly decreased (Fig. 8b–d), indicating that cell proliferation and differentiation were inhibited. Importantly, the addition of IL-1RA to HG-CM increased the proliferation and differentiation rates (Fig. 8b–d). By using MAb anti-mouse IL-1β to specifically block the IL-1β signal, the proliferation and differentiation of C17.2 cells also increased, and there was no significant difference compared with the IL-1RA group. These results indicate that IL-1RA and anti-IL-1β have similar protective effects against the damage caused by IL-1β. In summary, high glucose levels induce the release of IL-1β from microglia, which acts on NSCs, thereby impairing AHN.

When collating all of the above results, it appears that microglial NLRP3-dependent pyroptosis impairs AHN, which is achieved through the release of IL-1β. With the increase of NLRP3 expression, as caused by high glucose, microglial pyroptosis is aggravated and the release of IL-1β is increased, leading to impaired AHN and ultimately cognitive dysfunction. Down-regulating the expression of microglial NLRP3 can significantly improve AHN and alleviate cognitive dysfunction.

## Discussion

Diabetes mellitus is a chronic metabolic disorder that can exert destructive effects on multiple organs in the human body. Cognitive dysfunction is a common central nervous system complication of diabetes. Studies have indicated that cognitive deficits are present in patients with both type 1 and type 2 diabetes [35]. Identifying the pathogenesis of DE and developing potential therapeutic strategies is therefore of paramount importance. Our current results reveal that under hyperglycemic conditions, activation of the NLRP3 inflammasome in microglia leads to an increase in pyroptosis and IL-1β release. IL-1β exerts detrimental effects on NSCs, leading to the suppression of AHN and culminating in the cognitive impairments observed in the DE mouse model.

In our repeated animal modeling experiments, a specific pattern of body weight changes was observed: after multiple low-dose intraperitoneal injections of STZ in high-fat diet-fed mice, their body weight initially showed a brief decline, then gradually recovered to normal levels, and eventually exceeded the body weight of the Ctrl mice. This trend can be explained by the combined effects of STZ and the sustained impact of the high-fat diet: STZ has targeted toxicity toward pancreatic β-cells. Shortly after injection, it specifically damages pancreatic β-cells, leading to a significant reduction in insulin synthesis and secretion [36], which directly triggers metabolic disorders in the mice, resulting in temporary weight loss. Since the mice had previously been in a state of insulin resistance induced by a high-fat diet [37], and the pathological basis of compensatory hyperinsulinemia was not completely reversed, as the mice gradually adapted to the short-term damage caused by STZ, the regulatory effect of insulin resistance on body weight once again became dominant [28]. This ultimately led to a renewed increase in the body weight of the mice, exceeding that of the Ctrl group on a normal diet. In our study, DE mice exhibited impaired spatial learning and memory, aligning with previous observations in animal models of DE [30, 38]. Similar behavioral changes were observed in mouse models of Alzheimer’s disease (AD) [39, 40]. Accumulating evidences indicate that DE and AD share similar cognitive dysfunction and pathological changes [41], suggesting the possibility of a common pathogenesis. It is widely acknowledged that the hippocampus is a crucial brain region associated with cognition, learning, memory, and emotion. There is also evidence suggesting that individuals with diabetes exhibit hippocampal-related behavioral impairments, which are observed in both diabetic patients and animal models [42, 43]. NSCs possess the ability to proliferate and differentiate into various central nervous system cells, such as astrocytes and granule neurons which can integrate into the hippocampal circuitry to impact behavioral functions. This process is known as adult hippocampal neurogenesis [33, 44]. These adult-generated neurons are generated throughout life in the SGZ, and contribute to hippocampal-dependent functions, including learning and memory, stress regulation, and social behavior [45]. Moreno-Jiménez et al.'s study on brain tissues from AD patients at different stages revealed a significant and progressive decrease in DCX^+^ cells with disease progression [46]. Walgrave et al. showed that overexpression of miR-132 in adult mouse AD hippocampus can restore AHN and relevant memory deficits [47]. Mota B et al. found that a high-calorie diet in older rats impaired spatial learning and memory and increased anxiety, associated with a reduced number of DCX^+^ cells in the hippocampal DG [5]. The evidences above confirm the importance of AHN for hippocampus-related behaviors. In our study, there was a significant decrease in the proliferation and differentiation of NSCs in the DG region of diabetic mice, which was consistent with AHN impairment in the animal models of AD. These findings suggest that decline of spatial learning and memory functions in the DE mouse model may be associated with impaired hippocampal plasticity, while explaining the similarities in cognitive dysfunction as manifested in both DE and AD.

The role of neuroinflammation in cognitive impairment is well recognized. The hippocampus plays an important role in learning and memory and is particularly susceptible to the effects of neuroinflammation. As representatives of the primary immune effector cells in the central nervous system, microglia play a vital role in coordinating neuroinflammation through the secretion of immune mediators [48]. Our study focused on verifying that NLRP3-mediated pyroptosis and the release of IL-1β are associated with cognitive impairment in DE mice. Pyroptosis is an inflammation-related programmed death mediated by intracellular inflammasome and Gasdermin family member GSDMD, accompanied by the formation of cell membrane pores and the release of intracellular pathogens and pro-inflammatory mediators [49, 50]. Abnormal aggregation of inflammasome leads to the self-cleavage of pro-caspase-1 and the formation of active caspase-1-p20. Caspase-1-p20 cleaves GSDMD protein to form active N- and C-termini, with the N-GSDMD promoting cell membrane perforation and pyroptosis [51]. Caspase-1-p20 is involved in the maturation and release of the inflammatory cytokines IL-1β and IL-18 [52], which has also been confirmed in our research. Chronic hyperglycemia leads to mitochondrial dysfunction, endoplasmic reticulum stress, and oxidative stress. These pathological processes have been shown to induce the assembly and activation of inflammasomes, thereby promoting pyroptosis [53]. Our study found that the expression of key pyroptosis proteins were significantly upregulated in the hippocampal microglia of DE mice. Similarly, BV2 cells cultured under high-glucose conditions exhibited significantly increased expression levels of pyroptosis related proteins, along with elevated IL-1β and IL-18 secretion, providing corroborative evidence from this in vitro model. As the primary energy source for the brain, glucose is essential for maintaining the metabolic activity of immune cells and is a prerequisite for regulating immune responses. Consequently, the activation of microglia—the principal immune effector cells in the brain—is highly dependent on glucose availability. Numerous studies have demonstrated that exposure of microglia to high-glucose induces dose-dependent and time-dependent apoptosis [54, 55]. Additionally, it stimulates caspase-1 activity [56] and upregulates pro-inflammatory factors [57] in a dose-dependent manner. Consistent with these findings, our study confirms that high-glucose stimulation exacerbates neuroinflammation by inducing microglial pyroptosis in a dose-dependent and time-dependent manner.

A number of studies have reported that microglia, as immune cells unique to the central nervous system, can cause central inflammatory response and release of inflammatory cytokines after pyroptosis, thus promoting the progression of neurodegenerative diseases [27, 58]. Among different inflammasome cascades, NLRP3 inflammasome has received increasing attention in studies of innate brain immunity due to its relatively high expression in microglia [59]. Targeting NLRP3 inflammasome to inhibit microglial pyroptosis reduces spinal cord injury symptoms and neuroinflammation in intracerebral hemorrhage models [60]. Giving APP/PS1 AD model mice oral OLT1177—an inhibitor of NLRP3—restored spatial learning and memory deficits, while reducing microglial activation and the expression of pro-inflammatory cytokines [61]. To investigate overexpression of NLRP3 in microglia affects cognitive function by promoting microglial pyroptosis and increasing the release of pro-inflammatory cytokines in a DE mouse model, we conducted additional experiments by stereotactically injecting AAVs into the hippocampus to specifically modulate NLRP3 expression in microglia. With a reduction in NLRP3 expression in DE mice there were a recovery in cognitive function. Conversely, overexpression of NLRP3 in control mice impaired their learning and memory functions. When combining our results with that in the literature [42], robust evidence emerges indicating that activation of the NLRP3 inflammasome in microglia induces cognitive impairment in DE mice.

AHN is crucial for learning and memory, and neuroinflammation has been shown to be detrimental to AHN [33]. Previous studies have found that microglia, as important components of the neurogenic niches, participate in the regulation of AHN [16, 19]. Pyroptosis of microglia releases pro-inflammatory cytokines, including IL-18 and IL-1β. The detrimental effects of IL-1β on NSCs have been extensively studied in inflammatory diseases, with neuroinflammation observed to reduce the proliferation and differentiation of NSCs in both in vivo and in vitro experiments [62, 63]. With a targeted downregulation of microglial NLRP3 expression in DE mice, the expression of caspase-1-p20, N-GSDMD and IL-1β was reduced, accompanied by a significant increase in the number of precursor neural cells and immature neurons in the DG. Conversely, a targeted upregulation of NLRP3 in hippocampal microglia of control mice led to an increase in expression of pyroptosis related proteins, which in turn produced a marked reduction in the number of precursor neural cells and immature neurons in the DG. Corroborative effects were observed in our in vitro model. Here, C17.2 cell cultures treated with CM derived from high-glucose-treated BV2 cells and recombinant mouse IL-1β showed significantly decreased proliferation and differentiation rates.

Upon binding to IL-1R1 on NSCs, IL-1β activates intracellular signaling pathways, thereby regulating the transcription levels of target genes and promoting the cascade amplification of the inflammatory response [64, 65]. IL-1RA binds to IL-1R1 with an affinity nearly identical to that of IL-1β, thereby inhibiting IL-1β signaling [66]. We found that treatment with recombinant mouse IL-1RA ameliorated AHN impairment and restored cognitive function in DE mice. In vitro, application of IL-1RA or MAb anti-IL-1β restored the HG-CM-induced suppression of C17.2 cell proliferation and differentiation. The results demonstrate that NLRP3-mediated pyroptosis of microglia leads to a decline in cognitive function and neurogenesis, with IL-1β being an important driver of AHN impairment.

Our study has three of notable limitations. The use of AAV vectors with cell-specific promoters, rather than gene-edited animals specifically lacking microglial NLRP3, means we cannot rule out some off-target effects in the complex in vivo environment, thereby limiting a definitive microglia-specific attribution. Second, the experimental design did not include the Ctrl+AAV-shNLRP3 group and DE + AAV-NLRP3 group, which would have provided more complete causal evidence for the role of NLRP3. Additionally, the in vitro experiment was mainly based on the model system constructed by BV2 microglia cell line and C17.2 neural stem cell line. Although these cell lines have significant advantages such as standardized culture and high proliferation efficiency, it must be acknowledged that there are differences in physiological correlation between the immortalized cell lines and the primary cells [67], which may limit the biological significance of the study conclusion. Future studies will be required to address these issues.

In conclusion, we demonstrate that microglial NLRP3-dependent pyroptosis is increased in hippocampus of DE mice, which may inhibit AHN through the release of IL-1β, ultimately leading to cognitive deficits. A targeted downregulation of NLRP3 expression in microglia rescued AHN in DE mice by reducing IL-1β release, ultimately improving cognitive function. Therefore, these results provide important new information which can serve as the foundation for the development of NLRP-3 targets in the treatment of DE. Further research on the molecular mechanism will provide more comprehensive evidence for potential therapeutic applications of NLRP3 and to verify its possibility as a novel target for the development of drugs against DE.

## Supplementary information

Supplementary information

Supplementary fig 1

Supplementary fig 2

Supplementary fig 3

Supplementary fig 4

Supplementary fig 5

Supplementary fig 6
