# Supplementary material for: Microglial NLRP3-dependent pyroptosis promotes cognitive dysfunction of diabetic encephalopathy by inhibiting adult hippocampal neurogenesis through the release of IL-1β
Source: Acta Pharmacol Sin. 2026 Mar 20;47(8):2049–64. doi: 10.1038/s41401-026-01774-0 (PMC13388980; doi:10.1038/s41401-026-01774-0)
Supplement: Supplementary file 1 — Supplementary information [file 41401_2026_1774_MOESM1_ESM.docx]

**Supplementary tables**

**Table S1. List of the primary antibodies and secondary antibodies used in immunofluorescence.**

| **Name** | **Company** | **Product No.** |
| --- | --- | --- |
| Rabbit anti-NLRP3 | ABclonal | A5652 |
| Rabbit anti-caspase1 | ABclonal | A0964 |
| Rabbit anti-GSDMD | ABclonal | A18281 |
| Rabbit anti-IL-1β | ABclonal | A1112 |
| Goat anti-IBA1 | abcam | Ab289874 |
| Mouse anti-GFAP | Cell Signaling Technology | 3670 |
| Mouse anti-NEUN | Proteintech | 66836 |
| Rabbit anti DDDDK-tag (Binds to FLAG® tag sequence) | abcam | Ab205606 |
| Rabbit anti-Nestin | abcam | Ab221660 |
| Rabbit anti-SOX2 | abcam | Ab97959 |
| Rabbit anti-ki67 | abcam | Ab15580 |
| Rabbit anti-DCX | abcam | Ab207175 |
| Rat anti-BrdU | abcam | Ab6362 |
| Alexa Fluor 594 donkey anti-goat IgG | abcam | Ab150132 |
| Alexa Fluor 488 donkey anti-rabbit IgG | abcam | Ab150073 |
| Alexa Fluor 594 goat anti-rabbit IgG | abcam | Ab150080 |
| Alexa Fluor 594 donkey anti-rabbit IgG | abcam | Ab150064 |
| Alexa Fluor 488 donkey anti-mouse IgG | abcam | Ab150105 |
| Alexa Fluor 488 goat anti-rat IgG | abcam | Ab150157 |

**Table S2. List of primer sequences used in RT-qPCR**

| **Species** | **Primer name** | **Sequence (5’→3’)** |
| --- | --- | --- |
| Mouse | *NLRP3-F* | TCTGCACCCGGACTGTAAAC |
|  | *NLRP3-R* | CATTGTTGCCCAGGTTCAGC |
| Mouse | *β-actin-F* | CATCCGTAAAGACCTCTATGCCAAC |
|  | *β-actin-R* | ATGGAGCCACCGATCCACA |

**Supplementary Methods and Materials**

**BrdU injection and immunofluorescence staining**

BrdU (#B5002, Sigma-Aldrich) was dissolved in saline and protected from light. BrdU (50 mg/kg body weight) was administered via intraperitoneal injection for five days, twice every day at 8 h intervals. Tissue was collected 4 weeks after the final injection. The procedures for obtaining frozen brain sections and performing immunofluorescence staining followed the same protocol described in section “Methods and Materials - Immunofluorescence”. The special point is that for BrdU immunofluorescence staining, before blocking and primary antibody, DNA denaturation needs to be performed first. Frozen sections were treated with 1 M HCl at 37 °C for 15 min, followed by neutralization in 0.1 M borate buffer for 15 min.

**Supplementary figures**

Fig. S1 AHN is reduced in DG regions of DE mice.

(a) Representative confocal microscopic images of immunostainings and quantification for BrdU^+^/Nestin^+^ cells in DG regions of the hippocampus (*n* = 6). Scale bars, 100 μm. (b) Representative confocal microscopic images of immunostainings and quantification for BrdU^+^/SOX2^+^ cells in DG regions of the hippocampus (*n* = 6). Scale bars, 100 μm. (c) Representative confocal microscopic images of immunostainings and quantification for BrdU^+^/DCX^+^ cells in DG regions of the hippocampus (*n* = 6). Scale bars, 100 μm. The difference was analyzed using unpaired two-tailed *t* test. All data are shown as means ± SEMs. ****P* < 0.001, Ctrl vs. DE. Ctrl, Control group. DE, Diabetic Encephalopathy group.

Fig. S2. The AAV with F4/80-specific promoter has high microglial selectivity.

(a) Representative confocal microscopic images of Iba1 and Flag tag of AAV in DG regions. Scale bar, 50 μm. (b) Representative confocal microscopic images of GFAP and Flag tag of AAV in DG regions. Scale bar, 50 μm. (c) Colocalization analysis of Flag tag with Iba1 versus GFAP (*n* = 12 images per group). (d) Representative confocal microscopic images of NEUN and Flag tag of AAV in DG regions. Scale bar, 50 μm. The difference was analyzed using unpaired two-tailed *t* test. All data are shown as means ± SEMs. ****P* < 0.001.

Fig. S3. The levels of IL-18 in the supernatant of BV2 cells.

The levels of IL-18 in the supernatant of BV2 cells cultured with different concentrations of glucose or mannitol for 24 or 48 h, as measured by ELISA (*n* = 3 independent experiments). The difference was analyzed using two-way ANOVA. All data are shown as means ± SEMs. ^ns^ *P* > 0.05, **P* < 0.05, ***P* < 0.01, ****P* < 0.001, 5.5G group vs. other groups. G, Glucose. M, Mannitol. For example, 5.5G, 5.5 mM Glucose; 25M, 25 mM Mannitol.

Fig. S4. The treatment with IL-1RA ameliorates cognitive impairment in DE mice.

(a) Swimming speed in the MWM test (*n* = 6). (b) Escape latency during the MWM training period (*n* = 6). (c) Number of target crossings in the probe trail period (*n* = 6). (d) The time spend in target quadrant in the probe trail period (*n* = 6). (e) Representative motor trajectories on the learning period and the probe trail period in Ctrl and DE mice. The difference was analyzed using unpaired two-tailed *t* test or two-way ANOVA. All data are shown as means ± SEMs. ^ns^ *P* > 0.05, **P* < 0.05, ***P* < 0.01, and ****P* < 0.001, DE vs. IL-1RA. DE, Diabetic Encephalopathy group, IL-1RA, Diabetic Encephalopathy mice administered recombinant mouse IL-1RA.

Fig. S5. The treatment with IL-1RA alleviates AHN impairment in DE mice.

(a) Representative confocal microscopic images of immunostainings and quantification for Nestin^+^ cells in DG regions of the hippocampus (*n* = 6). Scale bars, 100 μm. (b) Representative confocal microscopic images of immunostainings and quantification for SOX2^+^ cells in DG regions of the hippocampus (*n* = 6). Scale bars, 100 μm. (c) Representative confocal microscopic images of immunostainings and quantification for ki67^+^ cells in DG regions of the hippocampus (*n* = 6). Scale bars, 100 μm. (d) Representative confocal microscopic images of immunostainings and quantification for DCX^+^ cells in DG regions of the hippocampus (*n* = 6). Scale bars, 100 μm. The difference was analyzed using unpaired two-tailed *t* test. All data are shown as means ± SEMs. **P* < 0.05, and ***P* < 0.01, DE vs. IL-1RA. DE, Diabetic Encephalopathy group, IL-1RA, Diabetic Encephalopathy mice administered recombinant mouse IL-1RA.

Fig. S6. The proliferation medium and differentiation medium respectively promote cell proliferation and differentiation.

(a) C17.2 cells showed increased proliferation in the proliferation medium. Scale bars, 500 μm. (b) C17.2 cells acquired a differentiated morphology in the differentiation medium. Scale bars, 500 μm. (c) Representative confocal microscopic images showing BrdU^+^ cells after different periods in proliferation medium (*n* = 5). Scale bars, 100 μm. (d) Representative confocal microscopic images showing ki67^+^ cells after different periods in proliferation medium (*n* = 5). Scale bars, 100 μm. (e) Representative confocal microscopc images of DCX⁺ cells cultured in proliferation or differentiation medium (*n* = 5). Scale bars, 100 μm. All IF images are representative of five independent biological replicates, with data from three technical images per replicate presented as the mean. The difference was analyzed using two-tailed *t* test. All data are shown as means ± SEMs. ****P* < 0.001.
