# Supplementary figures and images for: Microglial NLRP3-dependent pyroptosis promotes cognitive dysfunction of diabetic encephalopathy by inhibiting adult hippocampal neurogenesis through the release of IL-1β
Source: Acta Pharmacol Sin. 2026 Mar 20;47(8):2049–64. doi: 10.1038/s41401-026-01774-0 (PMC13388980; doi:10.1038/s41401-026-01774-0)

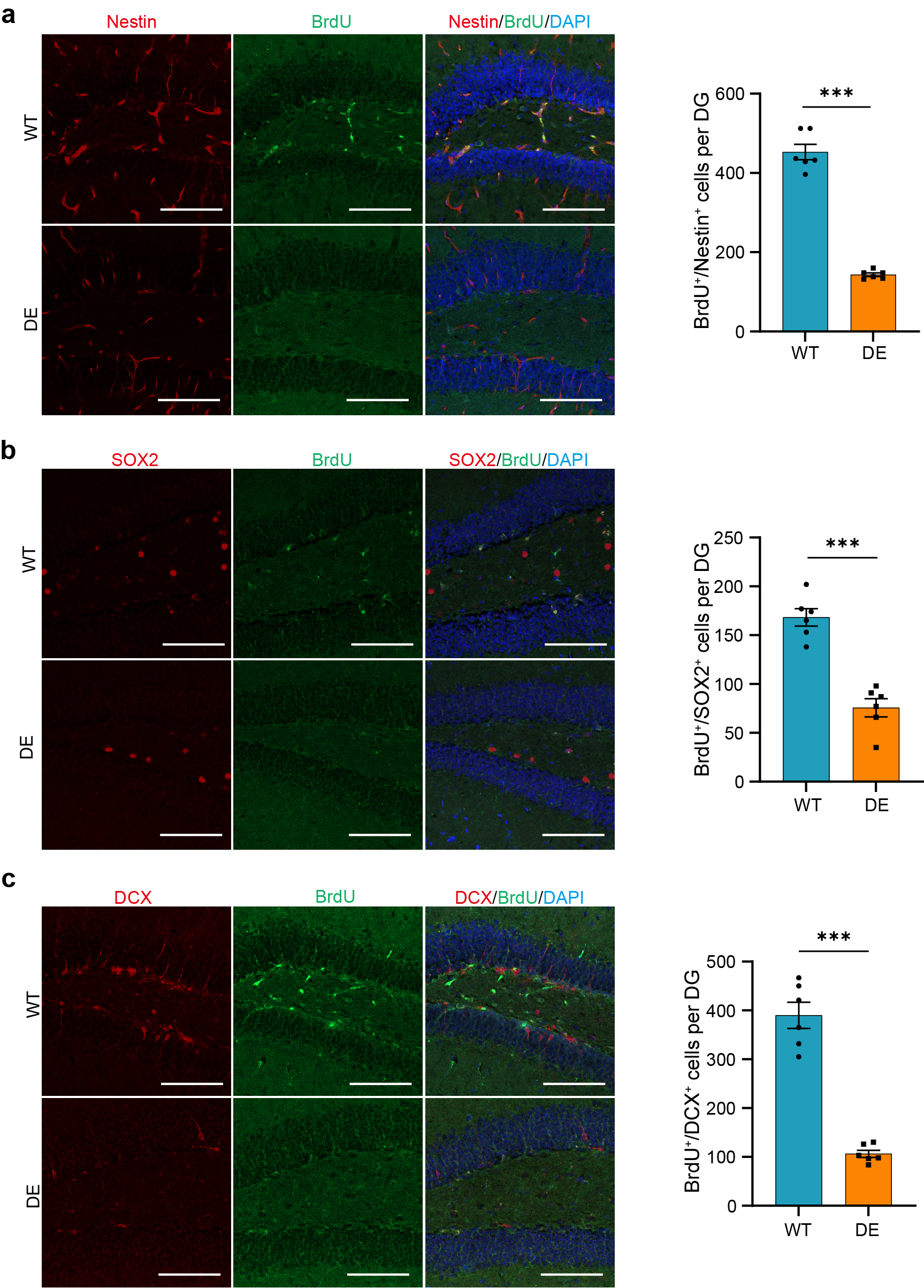

Supplement: Supplementary file 2 — Supplementary fig 1 [file 41401_2026_1774_MOESM2_ESM.jpg]

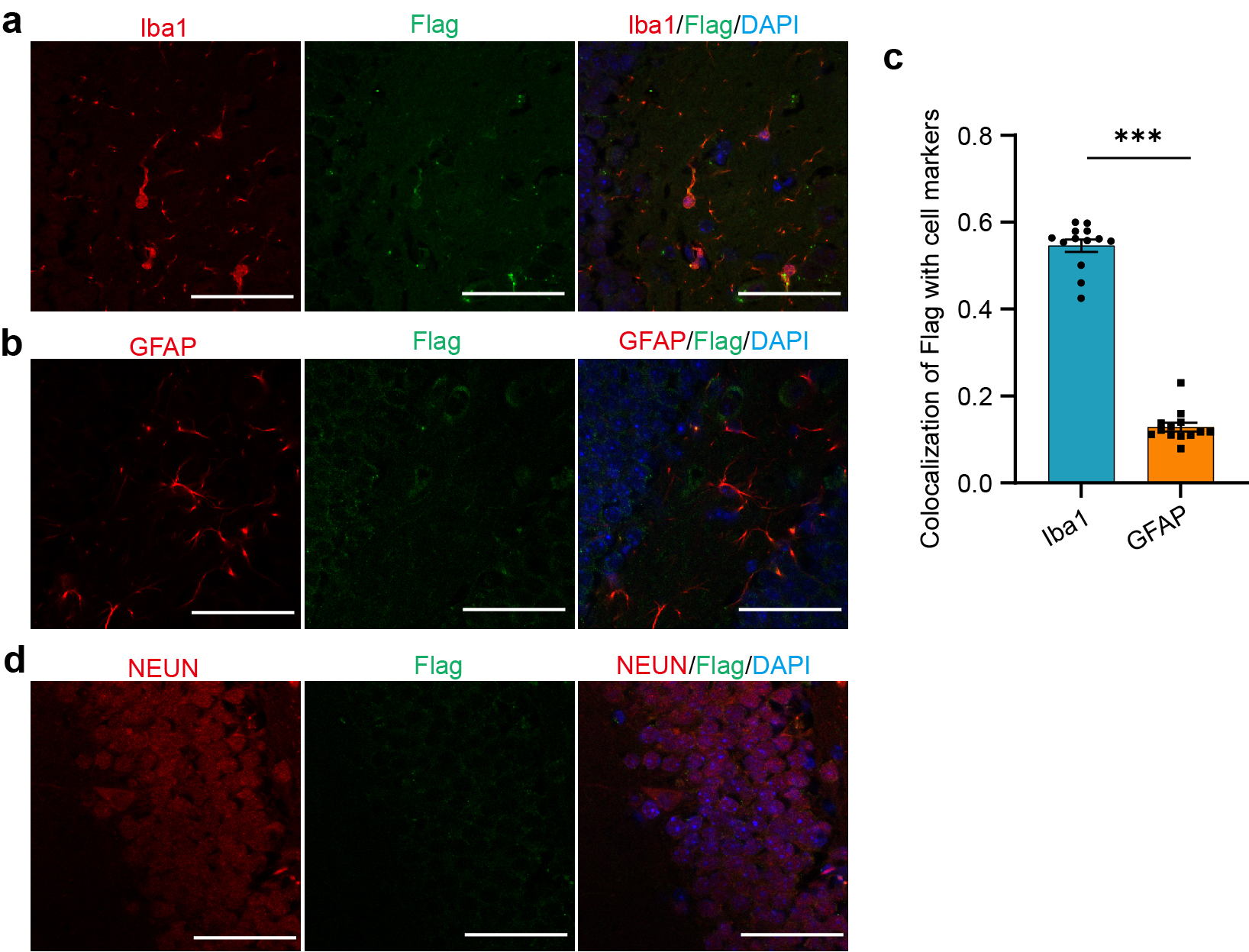

Supplement: Supplementary file 3 — Supplementary fig 2 [file 41401_2026_1774_MOESM3_ESM.jpg]

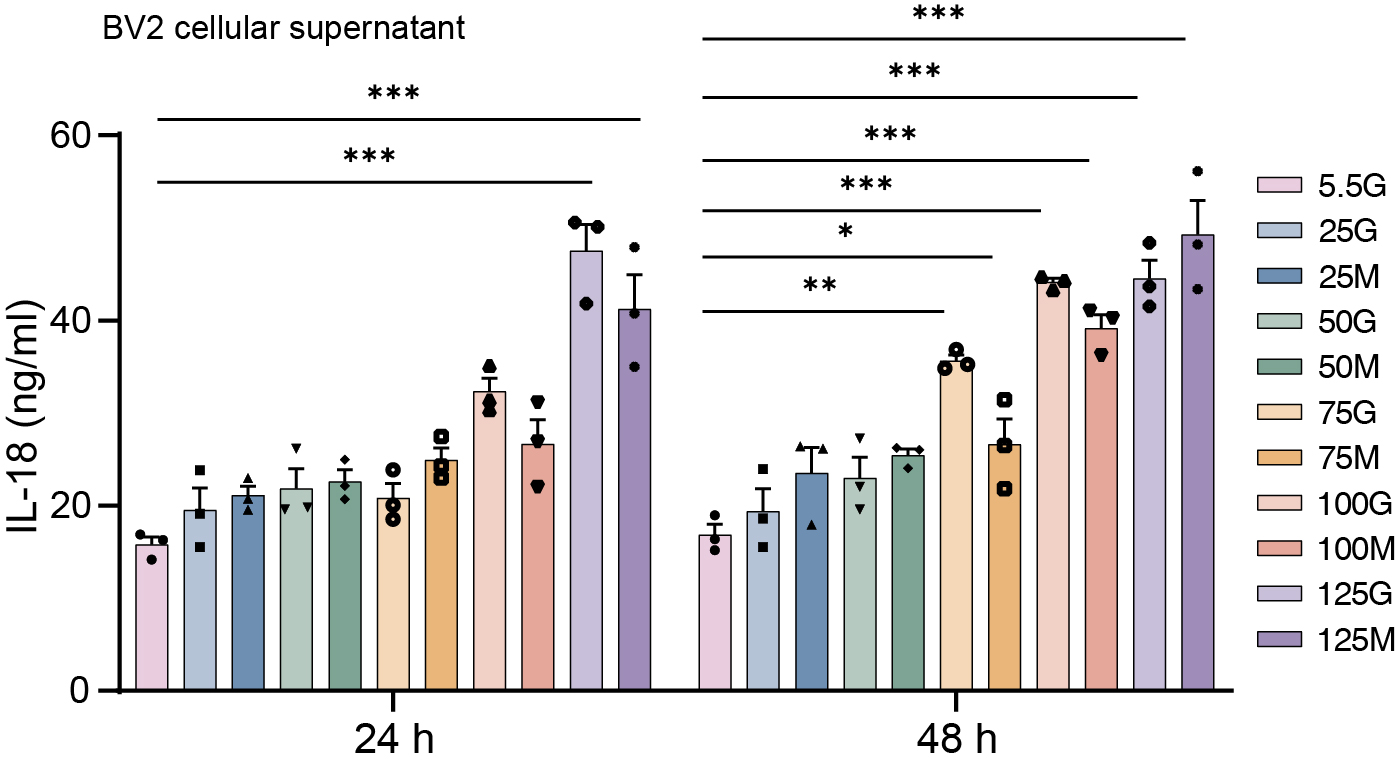

Supplement: Supplementary file 4 — Supplementary fig 3 [file 41401_2026_1774_MOESM4_ESM.jpg]

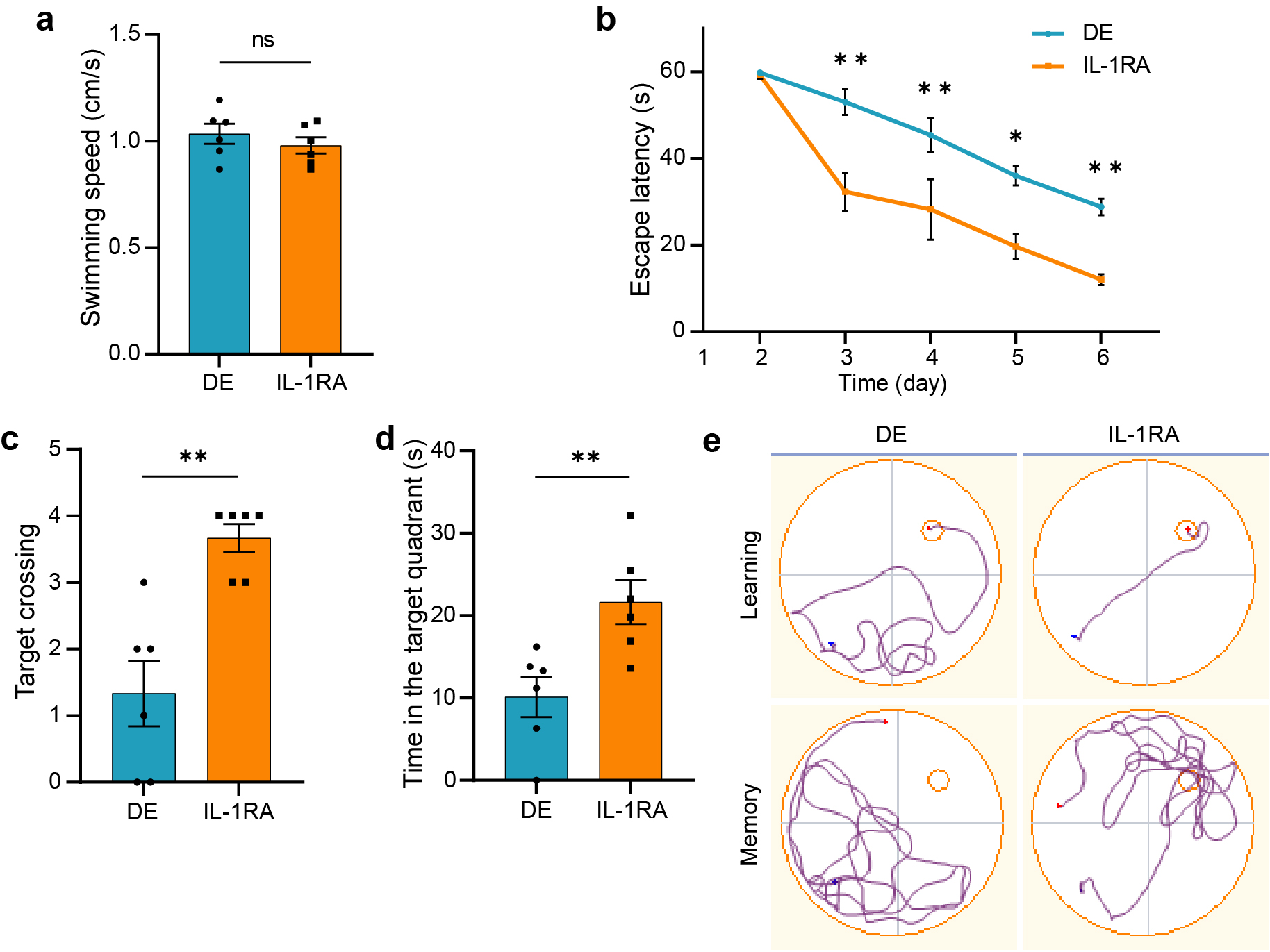

Supplement: Supplementary file 5 — Supplementary fig 4 [file 41401_2026_1774_MOESM5_ESM.jpg]

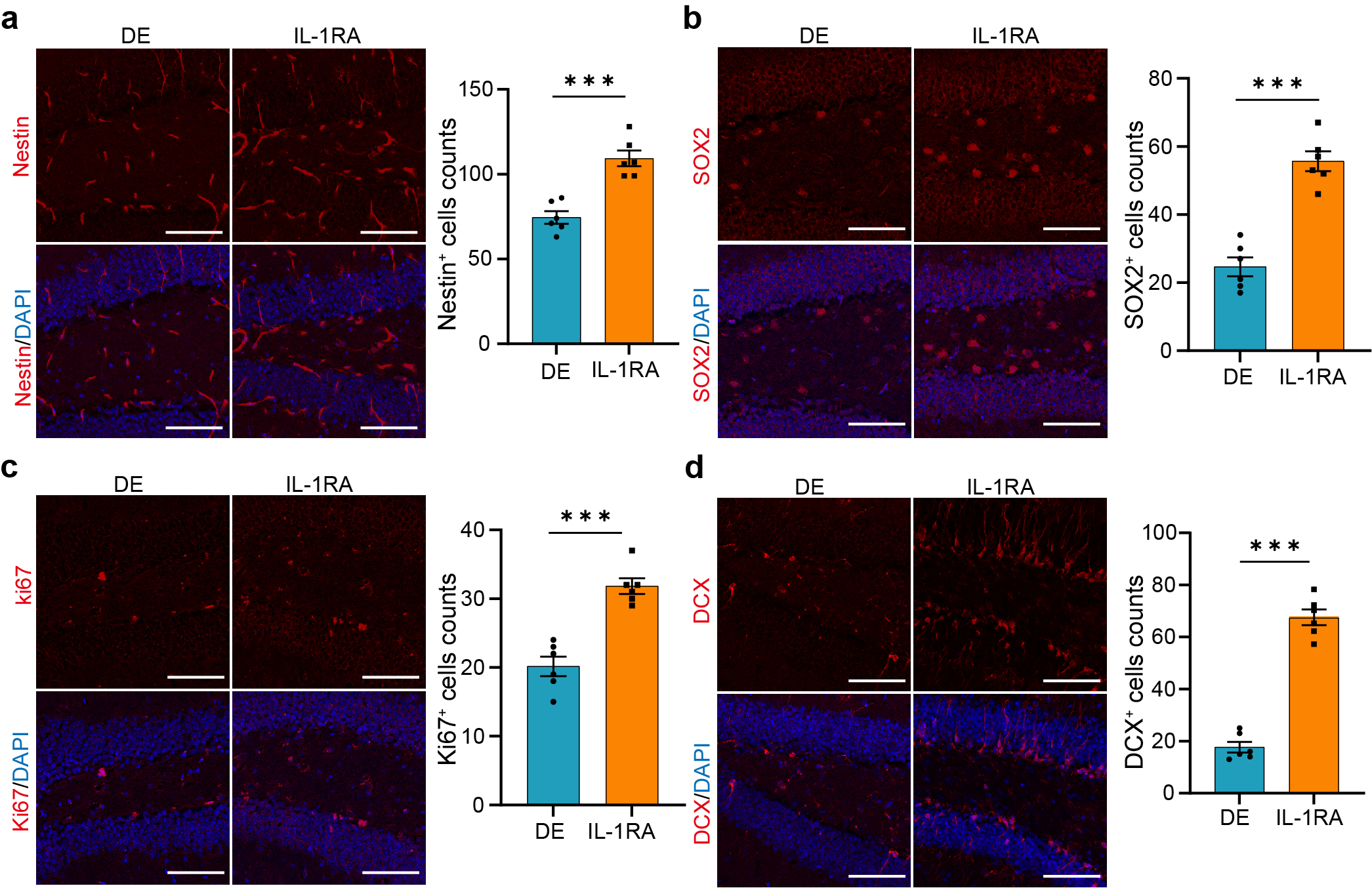

Supplement: Supplementary file 6 — Supplementary fig 5 [file 41401_2026_1774_MOESM6_ESM.jpg]

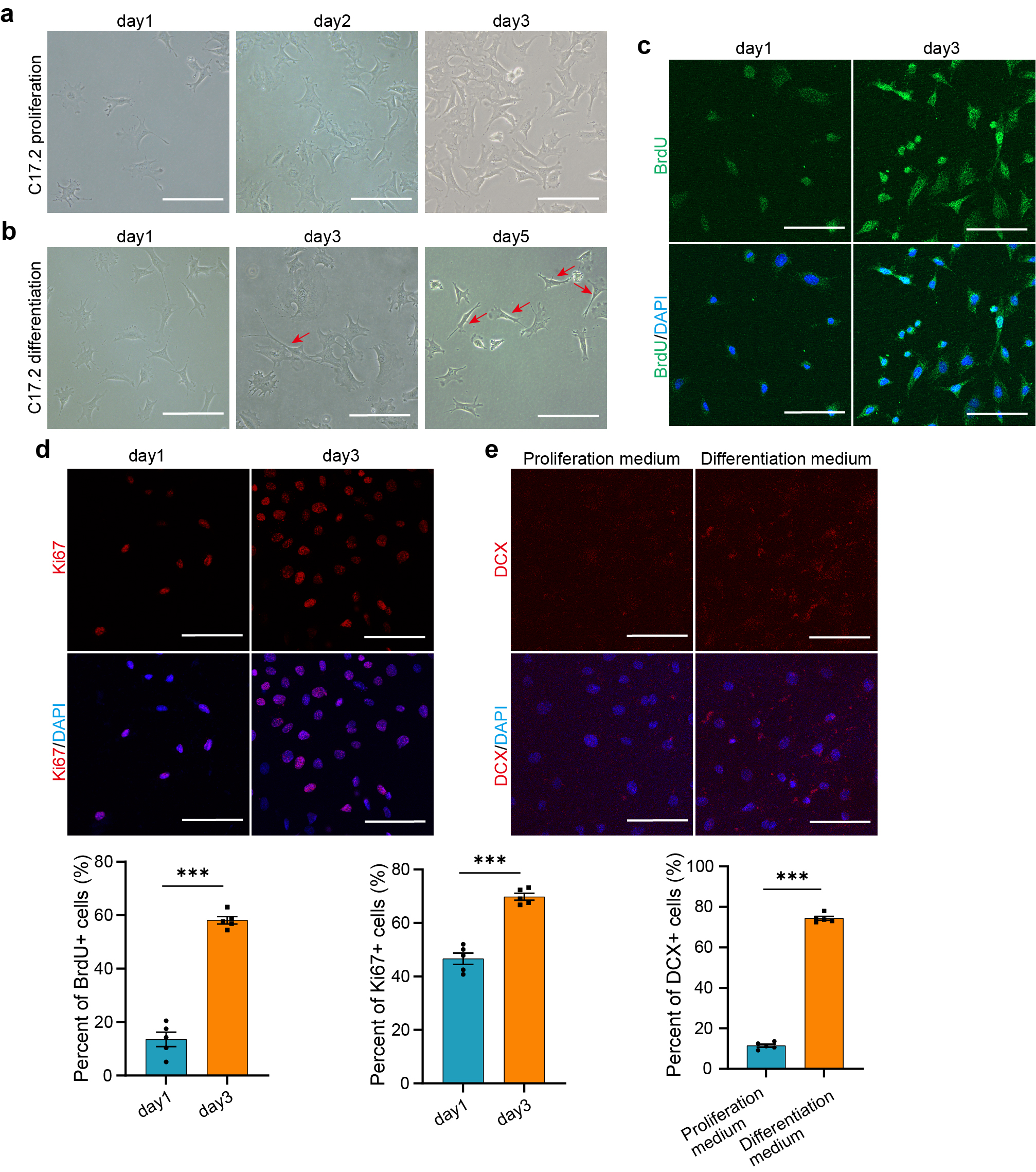

Supplement: Supplementary file 7 — Supplementary fig 6 [file 41401_2026_1774_MOESM7_ESM.jpg]
